# A well‐annotated genome of *Apium graveolens* var. *dulce* cv. Challenger, a celery with resistance to *Fusarium oxysporum* f. sp. *apii* race 2

**DOI:** 10.1111/tpj.70251

**Published:** 2025-06-09

**Authors:** Chaehee Lee, Lynn Epstein, Sukhwinder Kaur, Peter M. Henry, A. Dorien Postma‐Haarsma, J. Grey Monroe, Allen Van Deynze

**Affiliations:** ^1^ Department of Plant Sciences University of California Davis California 95616 USA; ^2^ Department of Plant Pathology University of California Davis California 95616 USA; ^3^ United States Department of Agriculture Agricultural Research Service 1636 E. Alisal St Salinas California 93905 USA; ^4^ Bejo Zaden B.V Trambaan 1A, 1749 CZ Warmenhuizen The Netherlands

**Keywords:** celery, breeding for disease resistance, *Fusarium oxysporum*, *Apium graveolens*, reference genome, nucleotide‐binding leucine‐rich repeat receptors, pattern recognition receptors, resistance gene analogs, genotype‐by‐sequencing

## Abstract

Celery (A*pium graveolens* var. *dulce)* production can be limited by the fungal pathogen *Fusarium oxysporum* f. sp. *apii* (*Foa*), particularly at temperatures above 22°C. Because celery has a narrow genetic base, an intraspecific admixture of *Apium graveolens* was developed into cv. Challenger, which is resistant to *Foa* race 2, the causal agent of *Fusarium* yellows, but susceptible to *Foa* race 4, a relatively unrelated causal agent of *Fusarium* wilt. We assembled a high‐quality, chromosome‐level physical map of Challenger with 40 464 RNA‐based, protein‐coding gene models in 3.3 Gbp and anchored it with a genetic map. Although there is high gene density and higher recombination at the ends of the chromosomes, an average of 56% of the genes/chromosome are in lower recombination zones (<0.025 cM/Mb). We identified Challenger's nucleotide‐binding and leucine‐rich repeat receptors (NLRs) and pattern recognition receptors (PRRs), the two gene families that encode most resistance (R) genes. In three treatment groups (mock‐infested or infested with either *Foa* race 2 or race 4), 243 NLRs and 445 PRRs were quantified in the celery crowns via Quant‐Seq 3′ mRNA‐Seq (Tag‐Seq). We compared the genomes of Challenger with that of the previously published cv. Ventura, which is moderately susceptible to *Foa* race 2. We present a toolbox for genome‐assisted breeding for celery that includes annotated gene models, a protocol for genotype‐by‐sequencing, documentation of the expression of NLRs and PRRs, and a straightforward strategy for introgressing selected NLR superclusters, 83% of which are in higher recombination regions.

## Significance Statement

“Western” celery (*A. graveolens* var. *dulce*) has a narrow genetic base and few genomic resources for breeding for critically important disease resistance. Here, we provide a Challenger genome with 40 464 RNA‐based, protein‐coding gene models that are anchored to a chromosome‐level genetic map, document the transcription of genes (243 NLRs and 445 PRRs) in the two major classes of resistance (R) genes in Challenger with and without the pathogen, and document a recombination landscape in *A. graveolens* in which an average of 56% of the genes/chromosome are in very low recombination zones (<0.025 cM/Mb).

## INTRODUCTION

Wild celery, *Apium graveolens* spp. *graveolens*, is native to wetter areas in the Mediterranean Basin, Europe, Asia Minor, and the Caucasus Mountains and southeastward toward the Himalayas (Lansdown, 2013). Historical records include the medicinal use of wild celery in Greece 2800 years ago and the introduction of plants into China during the Han dynasty (de Vilmorin, 1950). Rösch (2008) concluded that *A. graveolens* was cultivated, possibly as a spice, in gardens in southwestern Germany between the third and sixth centuries. Wild celery has been domesticated into at least three intraspecific crops: celery (*A. graveolens* var. *dulce*), which has enlarged and solid petioles; celeriac (*A. graveolens* var. *rapaceum*), which has an enlarged hypocotyl and upper taproot; and smallage, which is also known as cutting celery or leaf celery (*A. graveolens* var. *secalinum*). Smallage is eaten as either a leafy vegetable or a condiment or is used as a seed spice; it has foliage and generally hollow petioles that most resemble those of wild celery. “Chinese celery” or “local celery” has been called var. *dulce*, but is derived from an admixture of var. *secalinum* and var. *dulce* and has been classified as a separate group (Lai et al., 2025). “Western” celery (var. *dulce* sensu stricto) and probably celeriac were domesticated in Italy in the 17th century (de Vilmorin, 1950).

Celery is an important vegetable crop in the United States, China, and other parts of the world. In the United States, approximately 94% of celery is grown in California (https://quickstats.nass.usda.gov). In California in 2021, 748 522 metric tons of celery were harvested from 11 250 ha, with a value of $361 million. In the United States, celery has a very narrow genetic base, with most varieties derived from Giant Pascal (Quiros, 1993), which was first developed in France in 1884 (de Vilmorin, 1950).

There are thousands of strains of the fungus *Fusarium oxysporum*, many of which cause disease only in a single host. For example, *F. oxysporum forma specialis* (f. sp.) *apii* causes disease in celery, and indeed, *Foa* causes the most economically serious diseases of celery in the United States. In the early 20th century, the yellow or blanching celery varieties that were popular in the United States were susceptible to what is now called *Foa* race 1, which is a polyphyletic group (Epstein et al., 2017). In the 1950s, celery consumption in the United States changed to “green” varieties in the Giant Pascal lineage, which are resistant to *Foa* race 1. In 1976, *Foa* race 2, which is virulent in green varieties such as Tall Utah was reported in California (Hart & Endo, 1978; Otto et al., 1976); this is a single clonal lineage that is unrelated, that is, in a different clade of the *F. oxysporum* species complex (FOSC) than the *Foa* race 1 strains (Epstein et al., 2017). *Foa* race 2 was presumably subsequently transported on infested celery seeds (Epstein et al., 2022) to multiple states in the United States (Awuah et al., 1986), Canada (Cerkauskas & Chiba, 1991), Japan (Akanuma & Shimizu, 1994) and Argentina (Lori et al., 2016). In 1984, *Foa* race 3, which was also virulent on Tall Utah varieties, was identified primarily because it is vegetatively incompatible with *Foa* race 2 (Puhalla, 1984). However, there has been no evidence that *Foa* race 3 was ever economically important in celery production (Epstein et al., 2022). In 2017, a highly virulent *Foa* race 4, causal agent of *Fusarium* wilt of celery, was first reported (Epstein et al., 2017); *Foa* race 4 is in the same FOSC clade as *Foa* races 1 and 3. However, while *Foa* races 1 and 3 are avirulent on contemporary celery cultivars, *Foa* race 4 is virulent and generally highly virulent on all cultivars in temperatures that are conducive for disease (Henry et al., 2020).

As with many plant pathogens, resistance to a virulent *F. oxysporum* strain is often introgressed from wilder relatives of the crop (Chitwood‐Brown et al., 2021). Using classical breeding, Orton, Durgan, and Hulbert (1984), Orton, Hulbert, et al. (1984) transferred resistance to *Foa* race 2 from a reportedly celeriac into the celery breeding line UC1, whose exact parentage was unknown because there were multiple possible parents in the field in which the line was selected. Additional crosses of UC1 with the celery cultivar Tall Utah 52–75 resulted in UC390. The cultivar Challenger was developed by repeated selfing of the resistant line UC390. Currently, resistant cultivars such as Challenger are an important component of integrated pest management of *Foa* race 2 (Daugovish et al., 2008). However, cv. Challenger and other celery cultivars are susceptible to the new *Foa* race 4. Fortunately, progeny from a Challenger × *A. graveolens* PI 181714, which is probably var. *secalinum*, are resistant to *Foa* race 4 (Epstein & Kaur, 2023).

To accelerate celery breeding with modern techniques, more genomic resources are needed. Song et al. (2021) produced a chromosome‐level genomic assembly of celery cv. Ventura via Illumina short reads, 10X technology, and Hi‐C data, but publicly available gene models are limited. Ventura was developed in California and released in 1983 as a Tall Utah‐type cultivar (Quiros, 2016) with “field tolerance” to *Foa* race 2; that is, Ventura is less debilitated by race 2 than the named Tall Utah cultivars, but it is not resistant and is currently considered susceptible to race 2. Other *A. graveolens* assemblies include the following: a more fragmented assembly of cv. Baili, a Chinese celery (Cheng et al., 2022); a fragmented Illumina assembly of germplasm derived from cv. Jinnan Shiqin (Li et al., 2020), which is accession C176 in the Chinese celery group (Lai et al., 2025); and a chromosome‐level assembly of the celeriac cv. Alabaster (Lai et al., 2025). Some transcriptomic resources for *A. graveolens* are also available, for example, (Duan et al., 2020; Jia et al., 2015), but not for host–pathogen interactions. Thus, while progress has been made in developing genomic resources for *A*. *graveolens*, significant challenges persist, particularly in terms of genome assembly and gene annotation of celery, which are constrained by the quality and fragmentation of the assemblies and accessibility of annotations.

Resistance to *F. oxysporum* pathosystems in which the resistance (R) genes have been molecularly identified indicate that R genes are generally in one of two groups: (1) intracellular nucleotide‐binding and leucine‐rich repeat (NLR) groups of proteins with either coiled coil (CC)‐nucleotide‐binding (NB) leucine‐rich repeat (LRR) (CNL) or Toll/Interleukin‐1 receptor (TIR)‐NB‐LRR (TNL) (Chia & Carella, 2023) or (2) cell membrane‐associated extracellular pattern recognition receptors (PRRs) (Jones et al., 2024). In crops resistant to *F. oxysporum*, NLRs have been identified in five cases with three CNLs and two TNLs (Appendix S1). PRRs in the receptor‐like kinase (RLK) and receptor‐like protein (RLP) groups have also been identified as R genes in pathosystems with *F. oxysporum*. In three cases in tomato, R genes were introgressed from related species that encode for either an RLP in two cases or an RLK with a lectin domain (Gonzalez‐Cendales et al., 2016). In Arabidopsis, four R genes encode for RLKs, of which one has a lectin domain and one is in the wall‐associated kinase sub‐group (Hou et al., 2021; Huerta et al., 2023). A glutamate receptor‐like gene confers resistance to *F. oxysporum* f. sp. *vasinfectum* race 7 in cotton (Liu et al., 2021); whether it is a PRR *per se* is unknown.

Here, we developed foundational tools for breeding for *Foa* resistance in celery. Using various genomic data, including Pacific BioSciences HiFi (Menlo Park, USA), Iso‐Seq, Quant‐Seq 3' mRNA‐Seq (Tag‐Seq), and a genetic map, we assembled the cv. Challenger genome as a chromosome‐level reference genome and anchored the contigs to the genetic map. We provide high‐quality gene annotations with full‐length transcript support for 24 064 gene models and RNA support for the expression of an additional 16 400 genes. We bioinformatically selected members of NLRs and PRRs, the two gene families associated with the surveillance of plant pathogens, examined the expression levels of the NLRs and PRRs, and compared the DNA sequences in the Challenger cultivar with those in the *Foa* race 2‐susceptible cultivar Ventura. We used genotyping‐by‐sequencing (GBS) to confirm that *A. graveolens* PI 169001 is Challenger's *Foa* race 2‐resistant parent. Finally, we selected regions of interest in Challenger that may contribute to the improvement of other celery cultivars for disease resistance. The combination of Challenger's physical map, an *A. graveolens* genetic map, and extensive gene annotation indicates that the celery genome has two recombination landscapes; an average of 56 ± 4 (±SE)% of the genes/chromosome are in a very low recombination region (<0.025 centimorgans/Mb). The remainder of the genes are in the distal arms of the chromosomes, with an average recombination of 2.2 ± 0.2 cM/Mb, excluding telocentric chromosome no. 10 or 2.0 ± 0.2 cM/Mb/chromosome for the 11 chromosomes.

## RESULTS

### Celery cv. Challenger genome assembly and annotation

**Figure 1:**
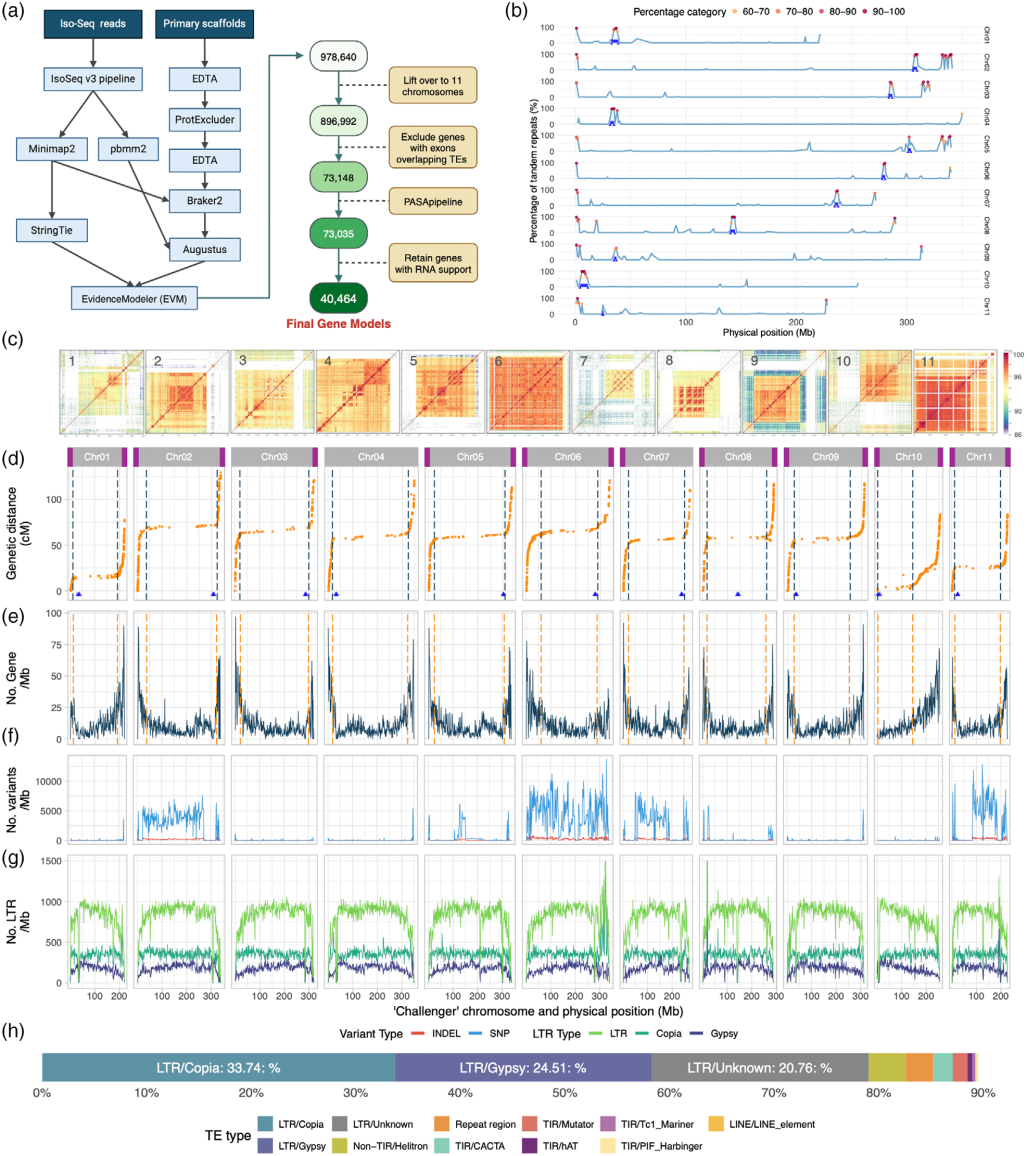
Features and annotation of the *Apium graveolens* celery cv. Challenger genome. (a) Overview of the genome annotation workflow with the selection of 40 464 gene models. (b) For each chromosome, the percentage of tandem repeats per 1 Mb window. The windows with different percent ranges >60% are marked in different colors. The blue triangles indicate the start and end positions of potential centromeres, highlighted by blue boxes. (c) Identity heatmaps displaying patterns of sequence identity of the centromeric region for each chromosome. (d–g) The *x* axes show the physical position by chromosome. Points on the *Y* axes are calculated for 1 Mb windows. (d) In the row where each chromosome is identified, a purple bar on the edge denotes the presence of a telomere in the assembly. The figures show a genetic map of *A. graveolens* in centimorgans and their corresponding physical map positions. The blue triangles on the bottom show the locations of the centromeres. The blue vertical dashed lines mark the boundaries of the lower recombination region. (e) Gene density. The red vertical dashed lines mark the boundaries of the lower recombination regions that were identified in panel d. (f) Density of heterozygosity, as measured by the density of SNPs in blue and indels in red based on PacBio HiFi reads. (g) Density of long terminal repeat (LTR) retrotransposons: green, total LTR; blue–green, Copia LTR; and dark blue, Gypsy LTR. (h) The major groups of transposable elements and repeat DNA in Challenger, from groups that account for the most bp to the least; LTR, long terminal repeats; TIR, terminal inverted repeats of transposable elements; and LINE, long interspersed nuclear elements.

Using 36X coverage from PacBio HiFi data, we achieved a reference‐quality *de novo* assembly of the Challenger genome, with a haploid length of 3.3 Gbp (Table 1, Figures S1). The genome annotation process (Figure 1a) was enriched with PacBio Iso‐Seq data from 15 diverse samples and Tag‐Seq data, which facilitated a high‐quality annotation that identified 40,464 gene models. Both the PacBio HiFi assembly (contig N50 = 162 Mbp) and the annotation are characterized by high Benchmarking Universal Single‐Copy Ortholog (BUSCO) scores (Table 1; Table S1), which demonstrate the quality and reliability of the data. One‐half of the length of the entire genome is in 8 contigs, and 90% of the length of the entire genome is in 32 contigs (Table 1). BUSCO analysis (Manni et al., 2021) revealed that of the expected genes in the cv. Challenger chromosome assemblies, 98.6% were present as complete copies, 0.4% were fragmented, and only 1% of the genes were absent (Table 1; Table S1).

**Table 1:** Descriptive statistics about the *Apium graveolens* cv. Challenger genome and its annotation

Genome assembly	*A. graveolens* ‘Challenger’	
	Primary assembly	Chromosomes
Number of sequences	2253	11
Size (bp)	3 428 536 321	3 267 747 404
Max length (bp)	303 905 413	349 664 189
N50 (bp)	162 067 254	320 415 245
L50	8	5
L90	32	10
Number of Gaps	—	52
%GC	36.6	35.7
BUSCO completeness (%)	98.6	98.6
Complete single‐copy (%)	91.6	93.4
Complete and duplicated (%)	7.0	5.1

Protein‐coding genes	Chromosome assembly
No. of genes	40 464
Total length of genes (bp)	154 876 985
% of genome covered by genes	5
Mean gene length (bp)	3827
Mean CDS length (bp)	1231
Mean exon length (bp)	279
Mean intron length (bp)	539
Mean number of exons per gene	5
BUSCO completeness (%)	97.3
Complete single‐copy (%)	92.8
Complete and duplicated (%)	4.6

RNA loci	Number	Total (bp)
tRNA	1000	76 298
rRNA	4292	1 274 972
miRNA	129	16 314
snoRNA	12 115	1 285 805

Previous studies have revealed that celery has 11 chromosomes (Iovene et al., 2008; Murata & Orton, 1984). To anchor the Challenger contigs, we employed two methods. Initially, leveraging the cv. Ventura assembly (Song et al., 2021), we assigned 95.3% of the Challenger sequence to the 11 chromosomes. Using our *A. graveolens* genetic map, which contains 11 linkage groups with a total of 1725 bins, we verified a 1:1 correspondence between the linkage groups and chromosomes. This genetic map, combined with read mapping coverage and the location of telomeric repeats, enabled significant enhancement of the accuracy of chromosome assembly, resulting in a reference‐quality celery genome with only 52 gaps (Table 1). The centromeric regions of all 11 chromosomes were demarcated and varied in length from 1 to 6 Mb (Figure 1b; Table S2). The selection of centromeric regions was supported by the high sequence identity in the heatmaps (Figure 1c). On the basis of the distribution of telomeric repeats at the ends of the chromosomes, eight of the 11 chromosomes are assembled end‐to‐end, three chromosomes are fully assembled on one end of the chromosome but not on the other, and only chromosome 4 is missing telomeres at both ends (Table [Link], [Link]).

For Challenger, we used the chromosome numbers that were established by Song et al. (Song et al., 2021). Celery karyotypes were previously characterized microscopically (Iovene et al., 2008) as 1 metacentric (here, identified as Chr08), 9 subtelocentric, and 1 telocentric (here, identified as Chr10). Challengers' chromosomes have an average length of 293 ± 14 Mb, with an average of 108 ± 5 cM (Figure 1d; Table [Link], [Link]). A comparison of the expressed SNP (eSNP) markers on the genetic map and the physical map revealed that for 8 of the 11 chromosomes, 83%–89% of the length of the chromosome had a reduced recombination rate of <0.025 cM/Mb. Chromosome 10 has the expected suppressed recombination at one end of the chromosome associated with a telocentric centromere. In contrast, in the distal arms of the chromosomes, there was an average recombination of 2.2 ± 0.2 cM/Mb, excluding telocentric chromosome no. 10 or 2.0 ± 0.2 cM/Mb/chromosome for the 11 chromosomes (Figure S3). Not surprisingly, there was a lower gene density in the region with a reduced recombination rate (Figure 1e). However, the regions with lower recombination rates still included 56% of the genes (Table S4).

The gene annotation pipeline detailed in Figure 1a identified 40 464 gene models (Table 1). All 40 464 gene models are supported by either Iso‐Seq or Tag‐Seq data, and their amino acid sequences for the models are provided in Data S1. These genes constitute 5% of the genome, with average lengths of 3827 bp for genes, 1231 bp for exons, and 539 bp for introns (Table 1). The gene models are high quality, with 1571 (97.4%) classified as complete BUSCOs out of 1614 genes from the Embryophyta_db10 database (Table S1). The distribution of gene density across the 11 chromosomes indicated that there was a greater gene density toward the chromosomal ends on each chromosome (Figure 1e).

Challenger has an overall heterozygosity of 0.26%. However, heterozygous loci are heavily concentrated on chromosome 6, intermediate on chromosomes 2, 7, and 11, and negligible on chromosomes 1, 3, 4, 9, and 10 (Figure 1f).

We identified over 3.6 million repetitive elements, accounting for 2.92 Gb and constituting nearly 90% of the genome (Table S5). Among these, the most prevalent were long terminal repeat retrotransposons (LTR‐RTs), which had a greater density in the lower recombination region than in the higher recombination region (Figure 1g; Table S6). Ty1/Copia and Ty3/Gypsy elements were the most abundant, comprising 33.7% and 24.5% of the genomic content, respectively (Figure 1h). LTR density is greater in the lower recombination regions than in the higher recombination regions; LTRs are significantly more skewed in their distribution than genes (*P*_Chi‐square_ < 0.0001) (Table S6).

### A GBS protocol confirms Challenger's *Foa* race 2‐resistant parentage and can track the diversity of *A. graveolens* accessions

**Figure 2:**
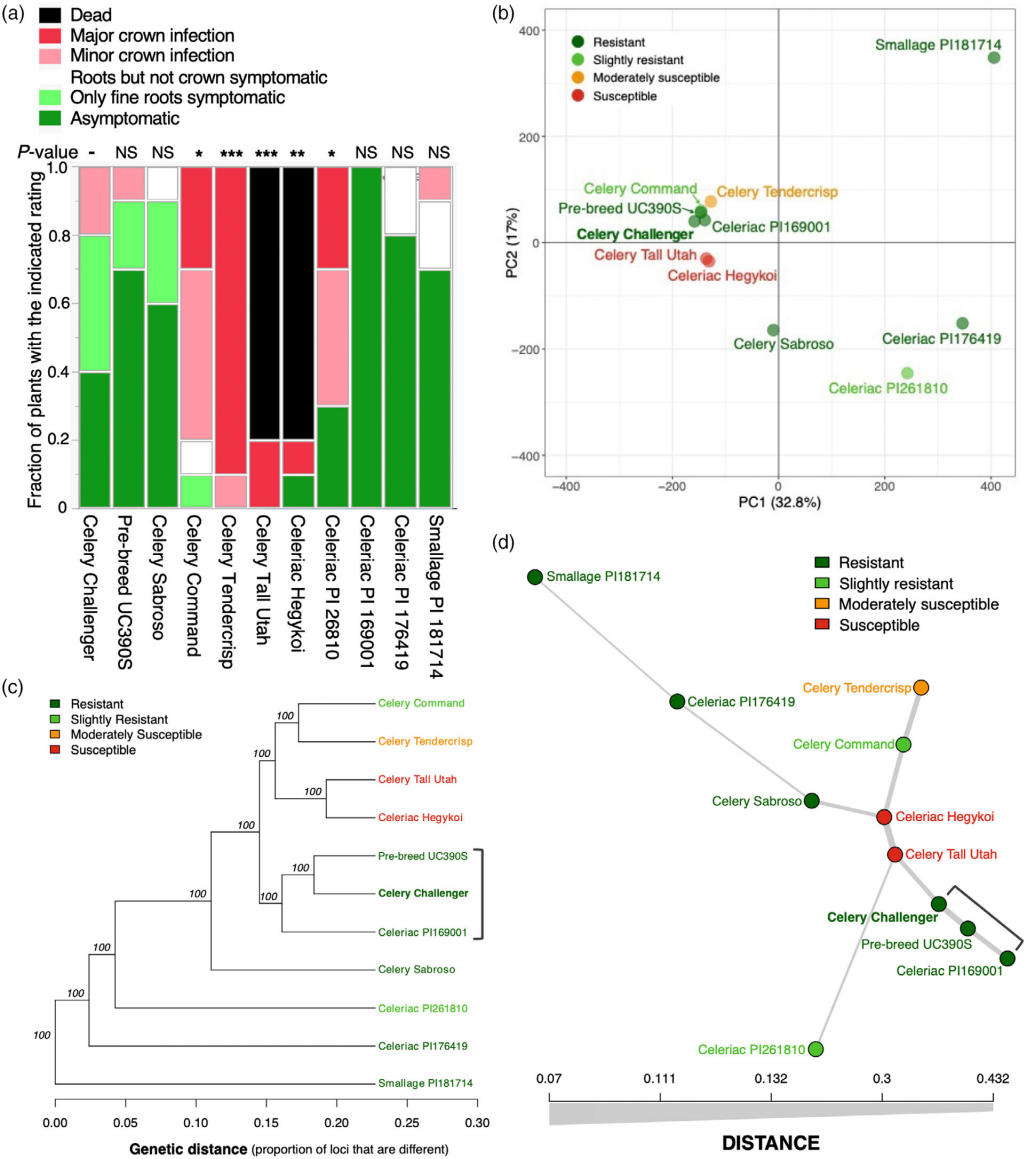
Analysis of 11 *Apium graveolens* accessions used for generating genotype‐by‐sequencing (GBS) SNPs. The accessions included five celery lines (*A. graveolens* var. *dulce*), one Challenger pre‐breeding line, four celeriac lines (var. *rapaceum* including PI 169001), and one smallage line (var. *secalinum*, PI 181714); the accessions were selected primarily to confirm the parentage of Challenger and are described further in Table S7. (a) Response of the accessions in an 8‐week greenhouse trial in *F. oxysporum* f. sp. *apii* (*Foa*) race 2‐infested soil (*n* = 10). The severity ratings of each accession were compared with those of Challenger's via the Steel nonparametric method. NS, not significant, *P* > 0.05. *P < 0.05; **P < 0.01. ***P < 0.001. There were also uninfested controls (*n* = 5, data not shown) that were all asymptomatic. (b–d) On the basis of Illumina sequences that were mapped onto the Challenger genome, over 2.16 million SNP markers were selected that differentiated between at least two of the accessions. Accessions are color‐coded by their response to *Foa* race 2. (b) Principal component analysis. PC1 through PC4 account for 73.4% of the variance. (c) Phylogenetic tree constructed via the unweighted pair group method with arithmetic mean (UPGMA) based on bitwise distances that were calculated from the variant data. The values at the nodes are the values of 100 bootstrap replicates. (d) A minimal spanning network plot. The length and thickness of the branches correspond to genetic distance.

Because the initial cross in Challenger's lineage of a *Foa* race 2‐susceptible celery × a *Foa* race 2‐resistant *A. graveolens* was performed with multiple accessions that were open‐pollinated in the field, we examined potential parents. Eleven *A. graveolens* accessions were selected (Table S7), tested for resistance or susceptibility to *Foa* race 2 (Figure 2a), and their DNA was processed for GBS. Filtered Illumina reads from each accession were mapped onto the Challenger assembly, and over 2.16 million SNP markers were identified. Historical field assays conducted in the 1980s (Orton, Hulbert, et al., 1984) were replaced with more precise greenhouse tests that indicated that only PI 169001 and PI 176419 have a similar level of resistance to *Foa* race 2 as Challenger. In a principal component analysis (Figure 2b), accessions did not cluster by resistance, as expected, for a trait encoded by one or a few genes. Additionally, phylogenetic analysis (Figure 2c) and a minimum spanning network (Figure 2d) demonstrated that of the potential race 2‐resistant parents, Challenger is most closely related to PI 169001. Based on the percentage identities of the SNPs of pairs of accessions, Challenger and PI 169001 share 85% identity, whereas Challenger and PI 176419 only share 44% identity (Table S8).

### Compared with celery cv. Challenger, Ventura is moderately susceptible to *Foa* race 2

**Figure 3:**
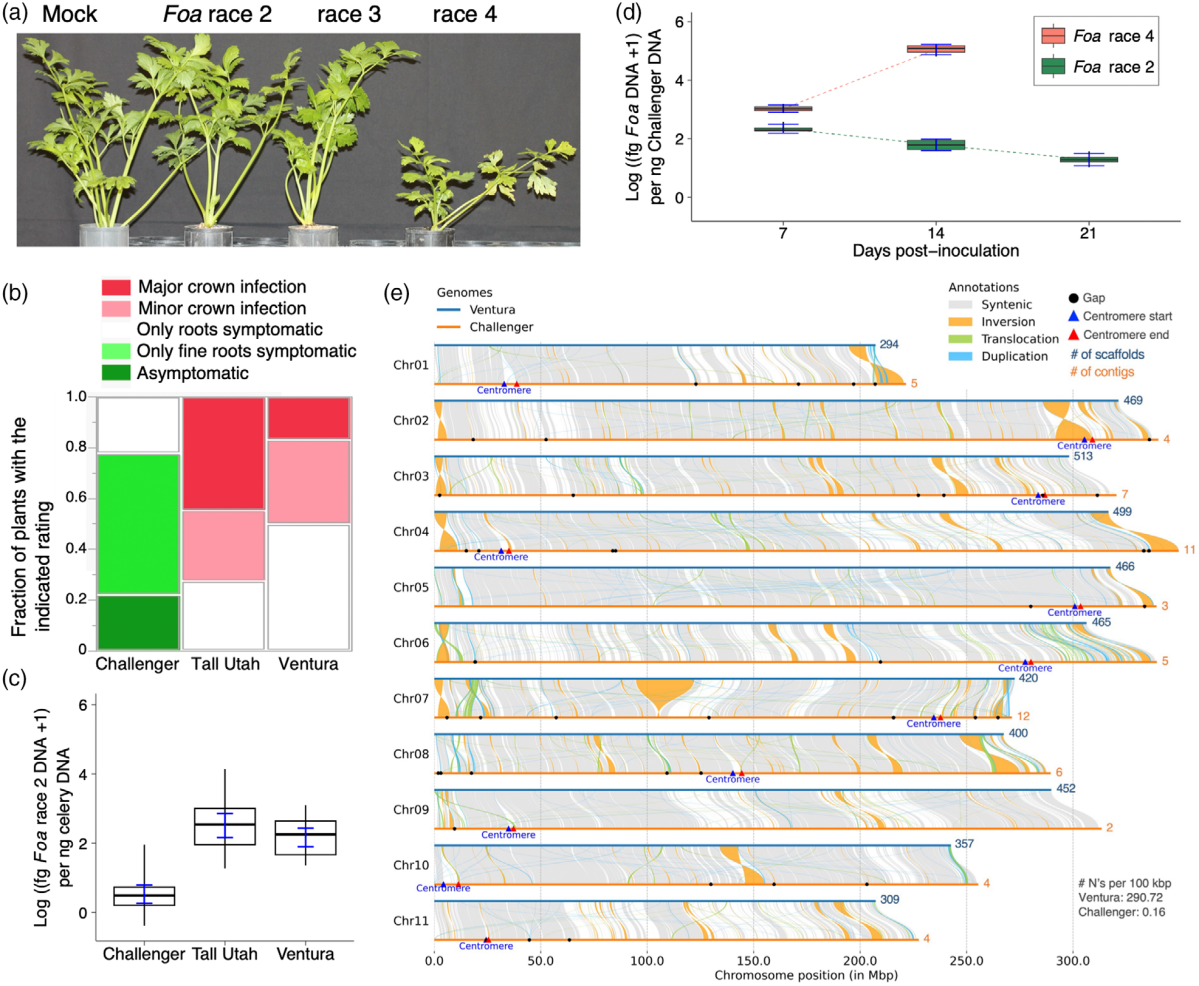
cvs. Challenger and Ventura: comparative *Foa* race 2 response and genomic architecture. (a) Challenger plants were grown either in (from left to right) uninfested soil or in soil infested with *F. oxysporum* f. sp. *apii* race 2 (*Foa*) race 2, *Foa* race 3 or *Foa* race 4. At 20 days post‐infestation (dpi), the median plant in stature was photographed. (b, c) In an independent experiment, a comparison of the severity of symptoms (b) and the log concentration (conc) of *Foa* race 2 in celery crowns (c) of three cultivars (Challenger, Tall Utah 52‐70R Improved, and Ventura) in an 8‐week greenhouse trial in soil that was infested with *Foa* race 2 (*n* = 18). Uninfested controls (*n* = 10) were asymptomatic (data not shown). (b) A nonparametric comparison for all pairs via the Steel–Dwass test indicated that the Challenger response was highly significantly different from the Tall‐Utah and Ventura responses (*P* < 0.0001) and that the Ventura and Tall‐Utah responses were not distinguishable (*P* = 0.19). (c) Box plots with error bars show the 95% confidence intervals. Whiskers extend from minimum to maximum value. None of the random subsamples (*n* = 4) of uninfested controls had any detectable *Foa* race 2 DNA in their crowns (data not shown). (d) A time course of the conc of either *Foa* race 2 or race 4 DNA in Challenger crowns in a separate trial shown as part a. Box plots with error bars show the 95% confidence intervals. None of the random subsamples (*n* = 4) of uninfested controls/timepoints had any detectable *Foa* DNA in their crowns (data not shown). (e) A comparison of the Challenger and Ventura genomes. Syntenic regions, which are connected by gray lines, had a minimum of 90% identity with a minimum alignment length of 100 base pairs (bp). The number of scaffolds for Ventura and contigs for Challenger is indicated at the end of each chromosome in blue and orange colors, respectively.

Challenger is resistant to *Foa* races 2 and 3 but susceptible to *Foa* race 4 (Figure 3a). The cultivars Ventura and the control Tall Utah 52‐70R Improved are susceptible to *Foa* race 2 (Figure 3b). Compared with Tall Utah and Ventura, Challenger had significantly less symptom severity (*P* < 0.0001, nonparametric Steel–Dwass) (Figure 3b) and significantly lower (*P* < 0.0001, ANOVA) *Foa* race 2 biomass in crown tissue (Figure 3c). Symptom severity and the concentrations of *Foa* race 2 biomass in crown tissue between Ventura and Tall Utah were not distinguishable (*P* = 0.19 and *P* = 0.25, respectively). In a separate trial that was also used for the Challenger Tag‐Seq analysis, the concentration of *Foa* race 2 DNA in celery crowns significantly decreased (slope = −0.08 ± 0.009, *P* < 0.0001) between 7 and 21 days post‐infestation (dpi) (Figure 3d). In contrast, in the compatible, Challenger – *Foa* race 4 interaction, the concentration of *Foa* race 4 increased significantly (*P* < 0.0001); between 7 and 14 dpi, the detransformed concentration increased by 106X. By 21 days, the *Foa* race 4‐infested plants had begun to die.

The genomes of Challenger and Ventura are syntenic, with a total of 2.18 Gb in syntenic regions (Figure 3e). However, 693.5 Mb (21%) of the Challenger genome had <90% identity with the Ventura genome, in a total of more than 10 000 segments (Figure S4). This discrepancy might be explained by the greater degree of fragmentation and consequently possible assembly inaccuracies in the Ventura genome than in the Challenger genome. Duplications were the second most prevalent structural variation between Challenger and Ventura, with 9128 duplications in Challenger that were single segments in Ventura; the greater number of duplications in Challenger is likely due to our enhanced resolution of repetitive elements in this genome with high‐resolution long‐read data. The assemblies also showed translocations and inversions; there were more translocations than inversions, but the affected regions in inversions were nearly twice as large as those impacted by translocations.

### Challenger's NLR family

**Figure 4:**
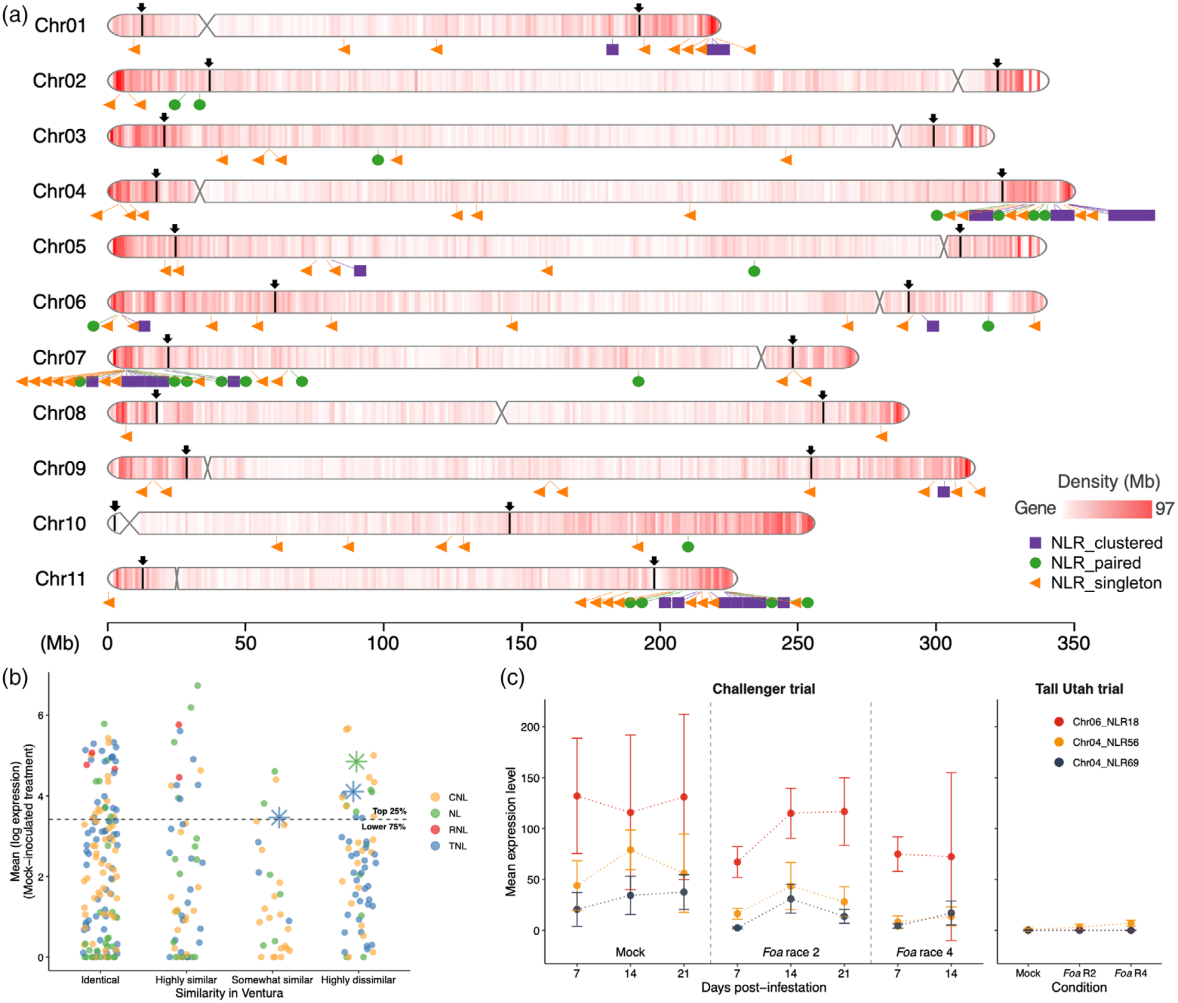
Celery cv. Challenger's nucleotide‐binding (NB) leucine‐rich repeat receptors (LRRs) (NLRs). (a) Diagram of Challenger's 11 chromosomes: gene density, boundaries of the lower recombination region and location of 287 NLRs. Inside each chromosome, gene density is shown on a scale from white (0 genes/Mb) to red (97 genes/Mb). Above each chromosome, the region between the two arrows and the extending dark line is the lower recombination region. The symbols below the chromosomes indicate the locations of the 287 NLRs. Purple squares indicate NLR clusters with three or more NLRs with no more than two non‐NLR genes among the NLR genes within the cluster. (b) Distributions of DESeq2‐normalized counts of 243 NLRs expressed in Challenger crowns in a mock‐infested treatment: 90 CC‐NB‐LRR (CNL); 42 NB‐LRRs without a 5′ domain (NL); 5 RPW8‐NB‐LRR (RNL); and 106 TIR‐NB‐LRR (TNL). Groups are based on the similarity of Ventura's homologous DNA to that of Challenger: identical in 100% of the length; highly similar, <100≥98% identical in 98% of the length; somewhat similar, <98≥75% identical in 75% of the length; and highly dissimilar, <75% identical in 75% of the length. Asterisks show the selections in part c. (c) DESeq2‐normalized expression levels with the 95% confidence interval of three selected NLRs in celery crowns in *Foa* race 2‐resistant Challenger, and in a separate experiment, race 2‐susceptible Tall Utah 52‐70R Improved (TU). After the plants were mock‐infested or infested with either *Foa* race 2 or *Foa* race 4, the Challenger crowns were harvested at the indicated time points and the TU were harvested at 21 dpi. The selections are in the upper quartile of expression of all NLRs, their DNA differs from that of their homologs in Ventura, and the homolog was not either not expressed or negibily expressed in TU: chromosome 4 NLR 69 (gene AgCh.04.g150590); chromosome 6 NLR 18 (AgCh.06.g193140); and chromosome 4 NLR 56 (AgCh.04.g150320).

An NLR was defined as a locus that contains an NBARC (NB) and at least two 3′ LRR domains. Two hundred eighty‐seven NLR loci were manually confirmed as either in an annotated gene or in an open reading frame (ORF) of a locus that was detected by NLR‐Annotator (Steuernagel et al., 2020). Among the 287 NLRs in Challenger, 116 are TIR‐NB‐LRR (TNL), 107 are CC‐NB‐LRR (CNL), 5 are RPW8‐NB‐LRR (RNL) and 59 are NB‐LRR (NL) without a 5′ CC, TIR, or RPW8 domain (Table 2; Data S2). We did not detect any additional integrated domains (NLR‐IDs) among the 287 NLRs. Figure S5 shows the number of NLRs of each type for each chromosome. Along the chromosome, NLRs are present as single genes, pairs of consecutive genes, or clusters with 3 or more NLRs with no more than two non‐NLR genes between NLR genes within the cluster (Figure 4a). Fifty‐eight percent of the NLRs are in clusters and 15% are in pairs. Chromosomes 4, 7, and 11 each have a “multicluster region” near the chromosome terminus (Figure 4a). Chromosome 4 has a 16 Mb multicluster with 60 NLRs, chromosome 7 has a 0.8 Mb multicluster with 25 NLRs and chromosome 11 has a 11 MB multicluster with 62 NLRs. Together, the three multiclusters account for 51% of the 287 NLRs. Two hundred forty‐two (84%) of Challenger's NLRs are in the higher recombination regions on the ends of the chromosomes (Table S6); there are only two small NLR 3‐member clusters in the lower‐recombination region in chromosomes 1 and 5. There is a significantly greater proportion of NLRs (*P*_Chi‐square_ = <0.0001) in the higher recombination regions than is the case for either total genes or BUSCO genes; total genes and BUSCO genes had similar distributions across lower and higher recombining regions (*P*_Chi‐square_ = 0.69) (Table S6).

**Table 2:** Similarity of NLR homologs between cv. Ventura and Challenger^a^

Category of similarity between Challenger's NLRs in Ventura	Criterion for category: identity (and length of alignment), (%)^a^	No. in each category (Expressed in Challenger (%)^b^)			
		TNLs	CNLs	RNLs	NL
Identical	100 (100)	59 (90)	51 (94)	3 (100)	28 (61)
Highly similar	<100≥98 (98)	21 (86)	16 (94)	2 (100)	15 (80)
Somewhat similar	<98≥75, (75)	4 (100)	19 (89)	—	6 (100)
Highly dissimilar	<75, (75)	32 (97)	21 (95)	—	10 (80)
Total		116 (91)	107 (93)	5 (100)	59 (73)

^a^ Two hundred eighty‐seven NLR loci in Challenger were manually confirmed as either in a bioinformatically annotated gene (*n* = 195) or in an open reading frame (ORF) of a locus (*n* = 92). Two hundred forty‐three NLRs were expressed in crown tissue, and an additional 11 were expressed in other tissues.

^b^ TNL, Toll/Interleukin‐1 receptor, nucleotide‐binding, leucine‐rich repeats; CNL, coiled coil, nucleotide binding, leucine‐rich repeats; RNL, *RESISTANCE TO POWDERY MILDEW 8* (*RPW8*) ‐ nucleotide binding, leucine‐rich repeats; NL, nucleotide‐binding, leucine‐rich repeats without a 5' domain.

### A comparison of Challenger's NLRs with their homologs in Ventura

Using the 116 TNLs, 107 CNLs, 5 RNLs, and 59 NLs that we documented in the Challenger genome (Table 2), we used BLASTn to identify homologs in the Ventura genome. Among the 287 total NLRs, while Ventura and Challenger had identical DNA sequences over 100% of the length of the gene model in 141 (49%) NLRs, Ventura had 63 (22%) with <75% identity over 75% of the length of the Challenger model. An additional 10% of Ventura's NLRs had between 75% and 98% identity in 75% of the length of the Challenger model. There is a greater proportion of NLRs in Ventura that are dissimilar to Challenger's gene models than the BUSCO genes (*P*_Chi‐square_ < 0.0001) (Table S9).

In a somewhat independent inquiry, we also used NLR‐Annotator to count NLRs in the DNA assemblies of the two cultivars. While Challenger had 119 TNL, Ventura had 14 fewer. Similarly, Challenger had 110 CNL, whereas Ventura had 21 fewer (Table S10). Notably, these differences were particularly apparent in the TNL‐dominated NLR multicluster on chromosome 4 and the CNL‐dominated NLR multicluster on chromosome 7.

### Expression of Challenger's NLRs in crown tissue

Two hundred NLRs (70%) of the 287 NLRs were confirmed via Iso‐Seq models; the Iso‐Seq samples were from crowns and 11 other tissues; of the 200 Iso‐Seq gene models, 189 were detected in Tag‐Seq samples from crowns (Data S2). The Tag‐Seq dataset also allowed monitoring of an additional 54 NLRs for a total of 243 NLRs that were expressed in crowns. The levels of expression of the 243 NLRs varied (Figure 4b; Figure S6). Although Challenger has only five RNLs, all five are expressed at a level that is in the upper quartile of NLR expression in crown tissue.

Because documented NLR R genes in various pathosystems are typically comparatively highly expressed (Brabham et al., 2024), we examined the NLRs that were in the upper quartile in expression in either the mock‐infested or infested with *Foa* race 2. We then selected the highly expressed NLRs that had homologs in Ventura that were dissimilar from those in Challenger; 17 NLRs in the upper quartile of expression had less than 75% identity in Ventura, and 4 had between 75% and 98% identity (Data S2). The 21 selected NLRs include 9 CNLs, 5 TNLs, and 7 NLs. Sixteen of these NLRs are in three locations: six of eight selected NLRs from chromosome 4 could be introgressed from a 9.15 Mb region from the terminus of chromosome 4 to NLR27; all six selected NLRs on chromosome 6 could be introgressed in a 24 Mb region (Chr6 NLRs 14–22); and all four selected NLRs on chromosome 11 could be introgressed in a 10.3 Mb region (Chr 11 NLRs 17–65). The five additional highly expressed NLRs in Challenger that are the most dissimilar to those in Ventura are located at the other terminus of chromosome 4 and on chromosome 7.

For each of the 287 NLRs for each time point, we used DESeq2 to determine whether the expression levels in Challenger crowns significantly differed across the selected treatments. *Foa* is a hemibiotroph; it is likely that *Foa* races 2 and 4 are in their biotrophic stages at 7 dpi and that *Foa* race 4 is in its necrotrophic stage at 14 dpi. There were no NLRs whose expression was significantly different between *Foa* race 2 and the mock group at 7 dpi. There were 16 NLRs whose expression levels significantly (*P*_adj_ < 0.05) differed between *Foa* race 2 and *Foa* race 4 at 7 dpi, with eight whose expression levels were lower in *Foa* race 4 than in *Foa* race 2 treatments, conceivably because a race 4 effector reduced NLR expression (Table S11).

### Potential NLR candidates for Challenger's resistance to *Foa* race 2

Because NLRs are often constitutively expressed (Von Dahlen et al., 2023) and we had no evidence of either up‐ or downregulated transcriptional regulation associated with either the pathogen treatments, dpi or the pathogen–dpi interaction, we mapped reads from a previous trial (Henry et al., 2020) in Tall Utah 52‐70R Improved (Tall Utah), which is susceptible to *Foa* race 2 and race 4. Tall Utah is an earlier variety than Ventura, and is presumably within Ventura's clade (Quiros, 2016). The Tall Utah trial was similar to the Challenger trial but differed in two aspects: (1) instead of dipping the crowns and roots in the inoculum, the plants were transplanted into infested soil, and the onset of disease was later than that in the Challenger trial, and (2) the crowns were only harvested at 21 dpi. Because the two trials were independent, we cannot compare the DESeq2‐normalized counts between the two trials. However, as an indication of presence/absence expression in the Ventura lineage, we used the Tall Utah dataset to look for those homologs that differed qualitatively, that is, expressed in Challenger and either not expressed or negligibly expressed in Tall Utah. Overall, log (DESeq2‐normalized expression +1) in Challenger and Tall Utah in the 21 dpi mock treatments were highly significantly correlated (*r* = 0.87, *P* < 0.0001). Among the 243 NLRs that were expressed in Challenger crowns, three were selected as potential candidates for *Foa* race 2 resistance (Figure 4c). All three NLRs are in the upper quartile of expression in Challenger and are either not expressed or negligibly expressed in Tall Utah. Chromosome 4 NLR 69 (AgCh.04.g150590) is a 5121 bp TNL in a cluster; its Ventura homolog has a SNP in the start codon and then a 5′ truncation that results in a frame shift mutation that results in many stop codons. In addition, there is a 3′ truncation in Ventura in the final exon that deletes a C‐JID domain. Chromosome 4 NLR 56 (AgCh.04.g150320) is a 5906 bp TNL; its homolog in Ventura has 5′ and 3′ truncations. While there is one alternative start codon, the 3′ truncation reduces the number of LRRs from 12 in Challenger to three. Chromosome 6 NLR 18 (AgCh.06.g193140) is a 3019 bp NL in a cluster. All three NLRs are located within the higher recombining regions and could be introgressed into a cultivar that is *Foa* race 2‐susceptible to determine if these particular NLRs or clusters confer *Foa* race 2 resistance.

### Challenger's PRR families and their expression in crown tissue

**Figure 5:**
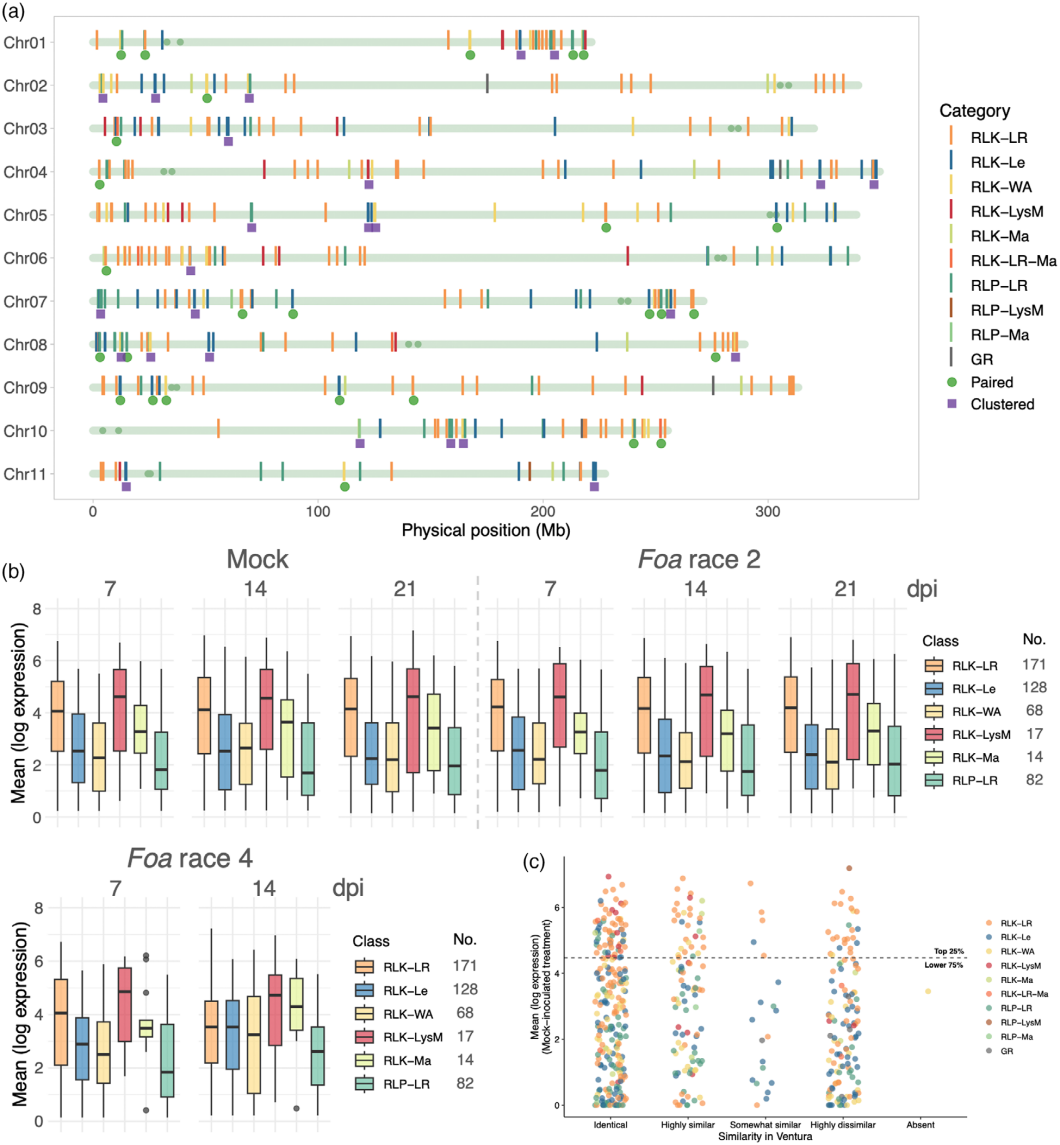
Potential pattern recognition receptors (PRRs) in Challenger. (a) Chromosomal locations of 494 gene models of PRRs. There are six subgoups with an intracellular receptor‐like kinase (RLK) with the following extracellular binding domains: RLK‐LR, leucine‐rich repeats; RLK‐Le, a lectin domain, typically for binding mannose; RLK‐WA, an intracellular wall‐associated kinase and typically a galacturonan binding domain; RLK‐LysM, with a chitin‐binding, LysM domain; and RLK‐Ma, with a malectin‐binding domain. There are three groups of intracellular receptor‐like proteins (RLPs) with the following extracellular binding domains: RLP‐LR, with leucine‐rich repeats; RLP‐LysM, with a LysM domain; and RLP‐Ma, with a malectin domain. GR, glutamate‐gated receptor. PRRs are designated pairs when there are two adjacent members in the same subgroup or clusters when there are three or more consecutive members in the same subgroup. Within the chromosome line, small circles represent the start and end of the centromeric region. (b, c). The plants were mock‐infested or infested with either *Foa* race 2 or *Foa* race 4 and then harvested at the indicated time points. (b) Box plots of log DESeq2‐normalized counts of 480 PRRs in Challenger crowns. The whiskers are 1.5 times the interquartile range (IQR) from the lower quartile to the upper quartile. (c) Distributions of DESeq2‐normalized counts of 494 PRRs in Challenger crowns: Categories are based on the similarity of Ventura's orthologous DNA to the Challenger's DNA sequence: identical, identical in 100% of the length; highly similar, identical in <100≥98% in 98% of the length; somewhat similar, identical in <98≥75% in 75% of the length; and highly dissimilar, <75% identical in 75% of the length.

We subdivided the receptor‐like kinases (RLKs) into six subgroups on the basis of the extracellular domains that could sense a PAMP or DAMP: RLK‐LR with leucine‐rich repeats; RLK‐Le with a lectin domain; RLK‐WA with a galacturonan‐binding domain; RLK‐LysM with a LysM domain, which typically binds chitin; RLK‐Ma with a malectin‐binding domain, which can bind particular peptides (Bellande et al., 2017); and RLK‐LR‐Ma with leucine‐rich repeats and a malectin‐binding domain. Similarly, we subdivided the receptor‐like proteins (RLPs) into three subgroups: RLP with leucine‐rich repeats (RLP‐LR), RLP‐LysM with a LysM domain, and RLP‐Ma with a malectin‐like binding domain (Table 3). Although PRRs (Figure 5a) are far less clustered than NLRs are (Figure 4a) and are less concentrated in the higher recombination region than in the lower recombination region compared with the NLRs, they are significantly more represented in the higher recombining region than either total genes or BUSCO genes (Table S6).

**Table 3:** Based on cell‐surface pattern recognition receptors in Challenger, the similarity of homologs in cv. Ventura^a^

Category of similarity between Challenger's PRRs and homologs in Ventura	Criterion for category: identity (and length of alignment), %^a^	No. in each category (expressed in Challenger, %)									
		RLK‐LR^b^	RLK‐Le^c^	RLK‐WA^d^	RLK‐LysM^e^	RLK‐Ma^f^	RLK‐LR‐Ma^g^	RLP‐LR^h^	RLP‐LysM^i^	RLP‐Ma^j^	GR^k^
Identical	100 (100%)	89 (93)	60 (90)	29 (97)	9 (89)	6 (100)	3 (100)	45 (73)	—	1 (0)	—
Highly similar	<100≥98 (98%)	33 (88)	16 (94)	17 (100)	6 (100)	5 (100)	—	20 (85)	—	2 (100)	—
Somewhat similar	<98≥75 (75%)	6 (83)	10 (80)	—	—	—	—	3 (100)	—	—	1 (100)
Highly dissimilar	<75 (75%)	43 (98)	42 (86)	21 (71)	2 (100)	3 (100)	2 (100)	14 93)	1 (100)	—	4 (75)
Absent^l^	—	—	—	1 (100)	—	—	—	—	—	—	—
Total		171 (93)	128 (88)	68 (90)	17 (94)	14 (100)	5 (100)	82 (80)	1 (100)	3 (67)	5 (80)

^a^ PRRs, pattern recognition receptors, are proteins on the plant cell surface that detect conserved patterns associated with either microbes or host cell damage. All the entries have either a transmembrane or a GPI anchor domain and a designated cell membrane localization by DeepLoc 2.1.

^b^ RLK‐LRs are iconic PRRs with an intracellular receptor‐like kinase and extracellular leucine‐rich repeat domains. Typically, Challenger's RLKs have a Pkinase_Tyr catalytic domain, and the LRR has LRRNT_2, LRR_1, LRR_6, and LRR_8 Pfam domains.

^c^ RLK‐Le contains an intracellular kinase domain and an extracellular lectin‐like receptor, which binds a specific carbohydrate. Fungal extracellular and wall proteins are typically mannosylated, and Challenger RLK‐Lec typically have an extracellular bulb‐type lectin (B_lectin) domain, which is also called a G‐type lectin S‐receptor‐like domain, which binds mannosyl residues. Typically, Challenger's RLK‐Le have a Pkinase_Tyr catalytic domain, a DUF3403 domain and a PAN‐2 Pfam domain. Approximately 8% of Challenger's lectins have an L‐type legB domain.

^d^ RLK‐WA, wall‐associated kinase containing an intracellular kinase domain and an extracellular galacturonan‐binding domain. Challenger RLK‐WA typically have a Pkinase_Tyr and a GUB_WAK binding domain. Many also have an EGF_CA‐binding domain.

^e^ RLK‐LysM have an intracellular kinase and an extracellular LysM domain, which bind chitin, a constituent of fungal walls. Some LysM domains also bind peptidoglycan oligosaccharides (Willmann et al., 2011).

^f^ RLK‐Ma contain an intracellular kinase domain (Pkinase_Tyr) and an extracellular malectin‐like domain. Malectin can bind to multiple cysteine‐rich peptides named rapid alkalinization factors (Franck et al., 2018).

^g^ RLK‐LR‐Ma contain an intracellular kinase domain and extracellular leucine‐rich repeats and malectin domains.

^h^ RLP‐LR, an iconic PRR with extracellular leucine‐rich repeats. Challenger RLP‐LR typically has LRRNT_2, LRR_1, LRR_6, and LRR_8 Pfam domains.

^i^ RLP‐LysM have an extracellular LysM domain.

^j^ RLP‐Ma have an extracellular malectin‐like domain.

^k^ GR, glutamate‐gated receptor. The genes have an ANF_(peptide) receptor, a Lig_chan, and an SBP_bac_3 domain. It is unknown whether GRs are legitimate PRRs.

^l^ Absent, no BLAST hits.

In the three treatments (mock‐infested, infested with either *Fo*a race 2 or *Foa* race 4), Challenger expressed 88% and 77% of their cell surface‐RLKs and RLPs, respectively, in their crowns: 155 RLK‐LR, 107 RLK‐Le, 59 RLK‐WA, 16 RLK‐LysM, 13 RLK‐Ma, and 5 RLK‐LR‐Ma, with a total of 355 cell surface receptor‐like kinases and 66 receptor‐like proteins, 63 of which are RLP‐LRs (Data S3). Figure 5b shows the variation in the levels of expression of log DESeq2‐normalized counts of the expressing RLKs and RLPs that have more than 10 members in the subgroup. Among the subgroups with fewer than 10 members, the range of expression of RLK‐LR‐Ma (*n* = 5) and RLP‐Ma (*n* = 2) is similar to that of RLK‐Ma (*n* = 13). The single RLP‐Lys (AgCh.11.g396090), which has two LysM domains, is comparatively highly expressed, with 964 ± 62 in the mock treatment at 7 dpi. For comparison, standard, single reference genes (Li et al., 2016) had the following counts in the mock treatment at 7dpi: ubiquitin c, 442 ± 10; β‐tubulin, 877 ± 56; and actin, 2029 ± 188. Among the 15 highest‐expressing Challenger PRRs in the *Foa* race 2 treatment at 7 dpi (above 500 DESeq2‐normalized counts), eight have identified orthologs in Arabidopsis; two RLK‐LysMs (AgCh.03.g077830 and AgCh.05.g161720) are orthologs of Arabidopsis AtLYK4 and AtLYK5, respectively; and in Arabidopsis, the two have overlapping functions in mediating a chitin‐triggered immune response. One RLK‐LR (AgCh.06.g193460) is an ortholog of SOBIR1, which is a positive regulator of defense but functions downstream of PAMP or DAMP detection (Liebrand et al., 2014).

Challenger has four glutamate‐gated receptors with a predicted localization in the cell membrane that were expressed in the crowns in all the treatments (Data S3). The ortholog of the glutamate receptor protein that is responsible for resistance in cotton to *F. oxysporum* f. sp. *vasinfectum* race 7 (Liu et al., 2021) is Challenger gene AgCh.10.g362560.

### Comparison of Challenger's potential PRRs with their homologs in Ventura

We used BLASTn to identify homologous PRRs in the Ventura genome. One RLK‐WA (AgCh.05.g178690) is apparently absent in Ventura, and 31% were classified as highly dissimilar (<75% identity in 75% of the length of the Challenger model) (Table 3). Among the other subgroups with >20 members, the Ventura homologs were highly dissimilar in 25% of the RLK‐LR, 33% of the RLK‐Le, and 17% of the RLK‐LR (Figure 5c). Similar to the NLRs, there were significantly more PRRs (*P*_Chi‐square_ < 0.0001) in Ventura that were dissimilar to Challenger's gene models than to the BUSCO genes (Table S9).

We did not detect upregulation of any PRRs in Challenger in the mock compared with the *Foa* race 2 group at 7 dpi (Table S12). However, Challenger had three PRRs that were significantly downregulated at 7 dpi; RLK‐WA (AgCh.08.g299320) counts decreased from an average of 105 in the mock group to 49 normalized counts in the *Foa* race 2‐treated group (*P*adj = 0.003); RLP‐LR (AgCh.03.g085400) counts decreased from 124 to 68 (*P*adj = 0.007) counts; and RLK‐Le (AgCh.04.g151100) counts decreased from 156 to 92 (*P*adj = 0.02) counts. In contrast, there were 41 PRRs whose PRR expression was significantly (*P*adj<0.05) lower in the *Foa* race 4‐treated group than in the *Foa* race 2‐treated group at 7 dpi (Table S12). There were also 54 PRRs in which *Foa* race 4 caused a significant increase in PRR counts.

## DISCUSSION

We present a high‐quality genome assembly and annotation of *A. graveolens* cv. Challenger, with 40 464 predicted protein sequences, which is similar to the recently published celeriac cv. Alabaster with 40 313 protein‐coding genes (Lai et al., 2025). Challenger is an open‐pollinated cultivar that is resistant to *Foa* race 2, the causal agent of *Fusarium* yellows of celery. Resistance is the preferred control method for celery infected with any strain of *Foa* and, indeed, for other crops that are infected with one of the hundreds of pathogenic strains in the *F. oxysporum* species complex (FOSC); historically, resistance to FOSC has been introgressed from a wild relative of the crop. Although new resistance‐breaking strains can appear within a FOSC clade via horizontal chromosome transfer of a pathogenicity chromosome (Ma et al., 2010) or by mutation of a pathogen avirulence gene (Biju et al., 2017), historically, resistance to FOSC has been reasonably durable; *Foa* races 2 and 4 are at least predominantly clonal and monocyclic, that is, reproduce only once per year.

*A. graveolens* has an average of only 0.37 ± 0.01 cM/Mb/chromosome in our mapping population, somewhat similar to other crop genomes with long chromosomes (Brazier & Glémin, 2022). While infrequent crossovers are expected in the region of the centromere, the regions of lower recombination, as defined here as <0.025 cM/Mb, extend well beyond the centromeric region in Challenger to 56% of the length of the entire pseudochromosome in telocentric Chr10, to a range from 68% to 89% of the lengths of the subtelocentric pseudochromosomes, and 84% of the length of metacentric Chr08 (Figure 1d; Table S4). Although Challenger has a greater density of genes in the higher recombination region (Figures 1d and 4a), it still has 56 ± 4% genes/chromosome in the region with lower recombination. Consequently, unless the extended suppression is an artifact from the two specific outcrosses that were used to prepare the genetic map, this lack of crossover and recombination presents several issues for breeding. First, if the gene of interest is from the lower recombination zone, there is a greater likelihood of linkage drag of genes from the non‐celery, resistant parent (Epstein et al., 2023; Rodgers‐Melnick et al., 2015). Second, celery is likely to have a greater incidence of deleterious mutations in the lower recombination zone. Third, if the genes of interest are not in higher recombination zones near the ends of the chromosomes, breeders will likely need to increase the number of progeny that are assessed for introgression of beneficial genes and loss of deleterious genes.

The genome of Challenger in this study is the highest quality celery genome published to date (Cheng et al., 2022; Li et al., 2020; Song et al., 2021). The final genome size is 3.26 Gb, which closely aligns with the 3.28 Gb estimated through k‐mer frequency analysis. The assembly is marginally smaller than that of cv. Ventura, which is 3.33 Gb (Song et al., 2021). Notably, although *A. graveolens* can outcross and Challenger is open‐pollinated, the celery genome has low heterozygosity at 0.26%, albeit slightly higher than Ventura's 0.20% (Song et al., 2021). Interestingly, both SNPs and INDELs were markedly greater in Challenger chromosomes 2, 6, 7, and 11, possibly reflecting either selective pressures or breeding practices that affected these regions.

In celery, nearly 90% of the genome is comprised of repetitive elements. While repetitive elements, particularly transposable elements, are responsible for the large genome size of most plants and crops, such as maize, wheat, and garlic (Hufford et al., 2021; Liao et al., 2022; Walkowiak et al., 2020), this proportion surpasses that observed in the maize genome, which constitutes 82% of its 2.1 Gb (Hufford et al., 2021), and closely approaches the repeat density found in one of the most repeat‐rich crops, garlic, which has 91% (Liao et al., 2022).

In the absence of pathogen pressure, plant breeding for maximal yield can lead to the loss of the full arsenal of genes that encode for pathogen surveillance, that is, the NLRs and PRRs that are important for genetic resistance (Barragan & Weigel, 2021). We have focused on resistance gene analogs in the NLRs, which are part of effector‐triggered immunity (ETI), and the PRRs, which are part of pattern‐triggered immunity (PTI); the PRRs extracellularly detect pathogen‐associated molecular patterns (PAMPs), microbe‐associated molecular patterns (MAMPs) and damage‐associated molecular patterns (DAMPs) (Zhang et al., 2024). Although the identification of R gene candidate(s) will require additional experimental evidence, such as bulk segregant analysis, the work here lays the groundwork for breeding for resistance to *F. oxysporum* f. sp. *apii*.

We annotated a total of 287 NLR genes in the Challenger genome, of which expression data in crowns supports the existence of 106 TNL, 90 CNL, 5 RNL, and 42 NLs. Previous work on celery genomes showed fewer NLRs, with only 62 reported in cv Ventura and 64 in celery cv. Baili (Liang & Dong, 2023; Song et al., 2021); based on Ventura, Song et al. suggested that celery is NLR‐deficient (Song et al., 2021). Although improvements in NLR annotation appear to partially account for the difference between the Challenger and Ventura NLR counts, our results are also consistent with cv. Ventura having fewer NLRs than Challenger. Based solely on NLR‐Annotator, Ventura has 14 fewer TNLs and 21 fewer CNLs than Challenger.

Future progress in understanding the role of NLRs in disease resistance in celery may be more rapid than that of PRRs, partly because celery NLRs are highly clustered and are located primarily in higher recombination regions at the ends of chromosomes. Indeed, introgression of three regions from Challenger in a total of 17.8 Mb could allow screening for resistance to *Foa* race 2 of 112 NLRs, including the three selections as shown in Figure 4c that are not expressed or negligibly expressed in Tall Utah but are highly expressed in Challenger. As a comparatively rapid strategy to determine if any of these 112 NLRs confer resistance to *Foa* race 2, any *Foa* race 2‐susceptible celery cultivar could be crossed with Challenger. We note that using the susceptible variety as the female parent has the advantage that any undesired selfs instead of F1 could be removed by screening the F1 for race 2 resistance, preferably in carefully controlled conditions for the most accurate phenotyping. Then a population of F1 could be selfed, and if informative PCR primers were selected, the F1S1 seedlings could be screened by PCR for the NLR zones of interest and tested for race 2‐resistance.

We note that NLRs are further divided into sensor NLRs (sNLRs), which include the CNLs and TNLs, and helper or executer NLRs, which include RNLs and NB‐LRRs (NLs) that do not have an iconic 5' domain (Ngou et al., 2022). When helper NLRs are in either an NLR pair or a cluster, they typically function downstream of the sensor NLR (Ngou et al., 2022). In their capacity as helper NLRs, NLs may reduce the biological costs of NLR resistance (Richard et al., 2018). However, NLs can have different functions in disease resistance. In Arabidopsis, an NL negatively regulates disease resistance via two neighboring sNLRs (Wu et al., 2022). In other pathosystems, for example, resistance in wheat to *Puccinia striiformis* f. sp. *tritici*, some NLs encode *bona fide* R genes (Zhang et al., 2019), sometimes without or with an extra integrated domain (NLR‐ID) (Michalopoulou et al., 2022); however, we did not detect any NLR‐IDs in Challenger.

Our selection for PRRs included bioinformatic screening for cell surface localization, that is, for attachment to the cell membrane with either a transmembrane domain or glycosylphosphatidylinositol (gpi) anchor; a transmembrane domain alone is insufficient to establish cell‐surface localization because proteins in intracellular membranes also have transmembrane domains. Consequently, we used DeepLoc 2.1 and NetGPI to confirm localization. However, our selection may be conservative because in some pathosystems, R genes are encoded by receptor‐like kinases that have a weak prediction for a transmembrane domain (e.g., *Rpg1* in barley) (Brueggeman et al., 2002) and are actually localized in intracellular membranes, the cytosol, and to a limited extent, in the cell membrane (Nirmala et al., 2006). In addition to using the software DRAGO3 to identify PRR candidates, we used our PFAM and eggnog annotations of Challenger to search for additional cell surface RLKs and RLPs, including wall‐associated kinases and kinase‐like (WAK and WAKL, respectively), and RLKs and RLPs with either malectin, LysM, lectin, or leucine‐rich repeat‐binding domains (Molina et al., 2024). We note that the binding domains may bind a broader range of PAMPs and DAMPS than indicated here; the Arabidopsis RLK‐LysM CERK1 binds chitin but also fungal and plant wall glucans and bacterial peptidoglycan (Yang et al., 2022). In addition, some of the PRRs, for example, the SOBIR1 orthologs, are involved in resistance signaling but not in pathogen surveillance *per se*.

The number of genes involved in resistance in Challenger and their progenitors has been difficult to assess (Orton, Durgan, & Hulbert, 1984), perhaps because, although celery is protandrous, it readily self‐pollinates, and complete emasculation of the flowers is difficult because the flowers are small (approximately 1 mm in diameter). Consequently, a population that is intended to be an outcross can be contaminated with selfs (Quiros, 1993). Nonetheless, one major and one minor gene in the UC1 pre‐breeding line were postulated to account for resistance to *Foa* race 2 (Quiros, 1993); this finding is consistent with the observation that Challenger is more resistant than Command both in the greenhouse (Figure 2a) and in the field (Table S7). A microscopic analysis of the celeriac parent of Challenger suggested that resistance was expressed in xylem parenchyma cells (Jordan et al., 1989), in keeping with results in other *F. oxysporum* pathosystems (Beckman, 2000). Here, the concentration of *Foa* race 2 DNA in Challenger crown tissue was greater at 7 days post‐infestation (dpi) than at 21 dpi (Figure 3d), which is consistent with *Foa* growing intercellularly in the cortex, even in a resistant host (Srivastava et al., 2024), and then containing the pathogen within the xylem‐associated cells. Notably, many *A. graveolens* accessions of var. *rapaceum* are resistant to *Foa* race 2 (Figure 2a; Table S13). However, whether race 2‐resistant accessions have the same or different R genes is unknown.

Here, we used GBS to confirm the source of resistance to *Foa* race 2 in Challenger and to test a protocol for selecting genome‐wide markers for *A. graveolens* accessions of interest. The GBS protocol is straightforward, partly because it uses a blunt‐end restriction enzyme that does not require any specific primers. Two of the older US celery cultivars (Tendercrisp and Tall Utah 52‐70R Improved) are considered to represent some of the diversity in a crop with a narrow genetic base (Lai et al., 2025; Quiros, 1993). Whereas the two celery cultivars have 81% identical SNP markers in our GBS study, there is a comparatively large diversity between some of the other accessions: for smallage PI 181714, there is only 38%–45% SNP identity with any of the other 10 accessions, and for two celeriacs (PI 1716419 and PI 261810), there is only 41%–55% SNP identity with any of the other eight *A. graveolens* accessions (Table S8). Recently, based on resequencing data, Lai et al. (2025) reported that PI 169001 is a smallage, rather than a celeriac, as it was listed in the USDA accession files.

All three domesticated *A. graveolens* are cool‐weather crops. In the United States, celery production is challenged by global warming, by *F. oxysporum* f. sp. *apii* races 2 and 4, and particularly by *F. oxysporum* when soil temperatures are in the 22–26°C range, which favors the pathogen and stresses the plant (Epstein et al., 2022). The accessions of wild *A. graveolens* ssp. *graveolens*, particularly from warmer climates in the Mediterranean where *A. graveolens* evolved, could be a source of germplasm with adaptations for both warmer climates and more diverse germplasm for disease resistance. While there are multiple collections of domesticated *A. graveolens*, there are few undomesticated accessions. Collections with wild *A. graveolens* are extremely limited; there is one collection of German accessions (Frese et al., 2018), but the southernmost latitude is only 47°.

## EXPERIMENTAL PROCEDURES

### Plants and fungi

Details about plant accessions are in Methods S1 and Table S7. *Foa* race 2 and *Foa* race 4 are clonal populations that are invariant in California within race (Epstein et al., 2017). Cultures of the isolates used here (race 2 isolate 207.A and race 4 isolate 274. AC) (Kaur et al., 2022) are available from the USDA‐ARS NRRL (https://nrrl.ncaur.usda.gov/).

### Challenger genome assembly

Challenger seeds were surface‐sterilized and incubated axenically on moist filter paper for 15 days. Approximately 600 axenic Challenger seedlings were frozen and used for isolation of high‐molecular‐weight DNA using a modified CTAB protocol (Stoffel et al., 2012) and made into a (long‐read) PacBio HiFi library using the manufacturer's protocol. The library was sequenced in four SMRT cells at the UC Davis Genome Center; we obtained a total of 124.1 Gb from 9.5 million reads, with an average read length of 13 kb (Table S14).

To estimate genome size, repetitiveness, and heterozygosity, the k‐mer frequency was calculated from PacBio HiFi reads via Jellyfish v2.2.10 (Marçais & Kingsford, 2011); the results were analyzed and visualized via GenomeScope v1 (Vurture et al., 2017). Owing to the high repetitiveness of the celery genome, in Jellyfish, we used 21‐mers with a maximum k‐mer depth of 1e6.

The PacBio HiFi reads were *de novo* assembled into contigs via Hifiasm v0.16.0 (Cheng et al., 2021), which was configured to target an estimated haploid genome size of 3 Gb. We first examined the primary assembly using synteny analysis with the Ventura genome using minimap2 (Li, 2018) and D‐GENIES (Cabanettes & Klopp, 2018). We then employed read mapping coverage and synteny analysis to identify and subsequently remove 415 small, duplicated contigs, which were erroneously duplicated in Challenger's primary assembly.

### Genetic map

Two populations were used for the genetic map: an *A. graveolens* var. *secalinum* x *A. graveolens* var. *dulce* with 3461 expressed SNP (eSNP) markers in 1146 bins and an *A. graveolens* var. *secalinum* x *A. graveolens* var. *rapaceum* with 1989 eSNP markers in 750 bins. All 1725 bins with 5261 genetic markers were aligned via BLAST to both the original 11 scaffolds and the updated assemblies (Camacho et al., 2009). The number of bins/chromosome ranged from 136 to 191. The length of the region of suppression of recombination in Figure 1d and Table S4 was estimated via a custom script that estimated the longest straight line with a slope <0.025 cM/Mb that had no points 2 cM from the line (https://github.com/lynnepstein/celery/).

### RNA isolation and sequencing

#### Iso‐Seq samples

A total of 15 samples were collected for PacBio Iso‐Seq (Methods S1, Table S15). After harvest, each of these tissues was quickly placed in prechilled aluminum foil, frozen in liquid N_2_, and then extracted using RNAeasy (Qiagen, Redwood City, USA) and indexed for one of two Iso‐Seq libraries.

#### Tag‐Seq trials

The Challenger trial was a completely randomized trial with three pathogen treatments (uninfested mock, infested with *Foa* race 2, and infested with *Foa* race 4) and either 2 or 3 sampling times; all the treatments were sampled destructively at 7‐ and 14‐days post‐infestation, but only the uninfested plants and those in the soil infested with *Foa* race 2 were sampled at 21 dpi because Challenger with *Foa* race 4 had started to die. Each replicate was a pool of 5 plants. Details are in Methods S1.

There was an independent greenhouse trial with cv. Tall Utah 52‐70R Improved. Methods were reported by Henry et al. (2020), Lohman et al. (2016). For each of the five replicates, eight celery cultivar Tall Utah 52–70 R Improved plants were either transplanted into uninfested soil or soil infested with either *Foa* race 2 or *Foa* race 4 and incubated for 21 days.

RNA was extracted and purified as described previously (Henry et al., 2020). Libraries were prepared by the UC Davis Genome Center via the Lexogen Kit and the manufacturer's recommendations (https://www.lexogen.com). In the Challenger and Tall Utah trials, a total of 110 and 77 Gb of sequence was obtained and analyzed as described previously (Jenner & Henry, 2022).

### Genome annotation

A nonredundant transposable element (TE) library was generated via Extensive de novo TE Annotator (EDTA) v.2.1.0 (Ou et al., 2019) on the primary scaffolds. To ensure that gene sequences were not inadvertently excluded during gene prediction, we used protExcluder v1.2 (https://www.canr.msu.edu/hrt/uploads/535/78637/ProtExcluder1.2.tar.gz) to remove any TEs in the nonredundant TE library that overlapped with sequences in the filtered protein database. The primary scaffolds were then reannotated with the updated TE library and softmasked for the downstream gene prediction pipeline. To identify TEs more precisely in the final Challenger genome, the same steps were performed independently again on the final 11 chromosomes.

To annotate the protein‐coding genes, we used minimap2 (Li, 2018) to align Iso‐Seq reads onto the softmasked primary scaffolds. We subsequently used brakaer2 v2.1.2 (Brůna et al., 2021) to generate a species‐specific training set for the gene prediction tools GeneMark‐EX and Augustus v3.1.0 (Keller et al., 2011). The initial *ab initio* gene prediction was performed via Augustus with collapsed transcripts and brakaer2 training data as extrinsic hints. We then used EvidenceModeler (EVM) v2.0.0 (Haas et al., 2008) to combine *ab initio* gene prediction and transcript alignment, generated by using StringTie V2.2.1, with different weights into consensus gene structures. The EVM gene models were transferred to the 11 chromosomes via liftoff v1.6.3 (Shumate & Salzberg, 2021). Because the number of final EVM gene models was unexpectedly high, we used bedtools v2.30.0 (Quinlan & Hall, 2010) to remove any models that had exons that overlapped with TEs. The remaining gene models were updated for different isoforms; the 5′ and 3′ UTR regions were added to the gene models via PASApipeline v2.5.2 (Haas et al., 2008). We then included an additional filtration step so that all the gene models were supported by either Iso‐Seq or Tag‐Seq RNA evidence. The last 500 bp regions of all the gene models that did not have Iso‐Seq support were blasted against all the Tag‐Seq reads (>2 read counts). Those gene models with Tag‐Seq support were included in our final gene models. The workflow of the gene annotation pipeline is provided in Figure 1a. The final gene IDs were curated via the modified python script GFF_RenameThemall.py (Morales‐Cruz et al., 2021). Protein and transcript sequences were retrieved via AGAT (Another GTF/GFF Analysis Toolkit) v0.9.165. The final gene models were functionally annotated with InterProScan 5 (Jones et al., 2014) and eggNOG‐mapper (Cantalapiedra et al., 2021). Gene Ontology (GO) terms were obtained from InterProScan.

rRNA genes were identified via Barrnap v0.9 (https://github.com/tseemann/barrnap#barrnap) using the Eukaryota database and categorized into 5S, 5.8S, 18S, and 28S rRNAs. miRNAs and snoRNAs were predicted via INFERNAL v1.1.470 with the Rfam 14.9 database. tRNA genes were searched via tRNAScan‐SE v2.0.1269 with eukaryotic parameters.

### Prediction of centromeric and telomeric regions

To examine the distribution of tandem repeats across the genome, we utilized the Tandem Repeat Annotation and Structural Hierarchy (TRASH) tool (Wlodzimierz et al., 2023) with 11 chromosomes and default parameters. We quantified the percentage of tandem repeats within 1 Mb windows to identify the regions enriched with tandem repeats, many of which are likely to correspond to potential centromeres. Specifically, windows displaying 100% tandem repeats were further investigated to delineate centromeric repeat units. Additionally, we employed ModDotPlot v0.8.4 (Sweeten et al., 2024) with the default parameter of window size (sequence length (bp)/2000 bp) to generate an identity heatmap for these centromeric regions. The identification of telomeric regions was corroborated by TRASH analysis and supplemented with manual verification to ensure accuracy.

### NLR and PRR selection

To predict NLR groups, we utilized NLR‐Annotator (Steuernagel et al., 2020) with default parameters and selected gene models that were functionally annotated with NLR‐associated domains. We then manually refined these NLR loci to ensure that they encompassed either a complete gene structure or an open reading frame (ORF), including the region annotated by NLR‐Annotator. The refined selection was restricted to loci that had a full complement of NLR‐associated domains, namely, CC‐NB‐LRR (CNL), TIR‐NB‐LRR (TNL), NB‐LRR (NL), or RPW8‐NB‐LRR (RNL). Because NLRs are frequently clustered or paired (Van De Weyer et al., 2019), we categorized the final NLRs into singletons, pairs (with no more than two non‐NLRs between any two NLRs), and clusters (comprising three or more NLRs with no more than two non‐NLRs between adjacent NLRs). The RIdeogram R package (Hao et al., 2020) was used to plot the gene density and distribution of NLRs across the 11 chromosomes.

PRRs were selected via two methods: (1) with DRAGO3 and then verification for cell surface localization with DeepLoc 2.1 (Ødum et al., 2024) and NetGPI 1,1 (Gíslason et al., 2021) or (2) keyword searches of PFAM terms (malectin, WAK, WAKL, LRR‐extensin, and lectin) followed by manual checking of domains and checking of cell‐surface protein localization as indicated above. The cutoff for Iso‐Seq expression was established at greater than zero on the basis of results from the IsoQuant tool (Prjibelski et al., 2023). For Tag‐Seq, support thresholds were set at values greater than 10, summing any conditions from either mock or *Foa*‐infested treatments.

### Genotyping‐by‐sequencing (GBS)

We selected celeriac USDA PI 169001, the presumed *Foa* race 2‐resistant ancestor of UC1 (Orton, Durgan, & Hulbert, 1984), and three possible but less likely ancestors: celeriac PIs 17 619, 261 810 and 320 912 (Table S7). We also included celery cv. Tall Utah 52‐70R Improved, which is a descendent of the presumed celery ancestor of UC1, Tall Utah 52‐70R Improved, and a less likely susceptible celery ancestor, Tendercrisp. As positive controls and to possibly assist with the selection of zones that might have markers for *Foa* race 2 resistance, we included two resistant relatives of Challenger: UC390S, a selection of UC390, which is the parent of Challenger, and cv. Sabroso, a cultivar for celery “sticks,” which was derived from UC1. We also included celery cv. Command (Trammell & Pybas, 2007), which has UC1 heritage via cv. Matador but is more horticulturally desirable but less *Foa* race 2 resistant. Celeriac has both *Foa* race 2‐susceptible and resistant cultivars, and here, PI 320912 represents a susceptible celeriac. To procure DNA, the seedlings were grown in a greenhouse. The fourth leaves of the plants were lyophilized, ground in liquid N_2_ in a mortar and pestle and purified via the Macherey‐Nagel Nucleospin Plant II Kit following the manufacturer's protocol, except that the RNase step was extended to 30 min. DNA was digested with the blunt‐end cutting restriction enzyme MlyI using the Hill et al. GBS protocol (Hill et al., 2023). Sequencing libraries were constructed with an average size of 300–350 bp (Monson‐Miller et al., 2012).

The quality assessment of the GBS data for the 11 *A. graveolens* accessions was performed via FastQC v0.11.9 (Andrews, 2010). After the raw reads were polished via Trimmomatic v0.39 (Bolger et al., 2014), the trimmed reads were mapped to the Challenger genome via BWA v0.7.17 (Li, 2013). Variant calling was conducted via the MultisampleVariantsDetector module from NGSEP 4 (Tello et al., 2023) with default parameters. The resulting VCF file was filtered to retain the biallelic variants called from all 11 samples with depths greater than one. Using the R package vcfR (Knaus & Grünwald, 2017), with the incorporation of resistance and susceptibility data, we performed principal component analysis (PCA) and minimum spanning network (MSN) analysis. Additionally, we constructed a phylogenetic tree via the unweighted pair group method with arithmetic mean (UPGMA), which is based on bitwise distances calculated from the final variant data. The tree was generated with 100 bootstrap replicates to assess node support, with a bootstrap cutoff of 50% to ensure reliable clade formation.

### Assays for symptoms and either *Foa* biomass in tissue

The bioassays for *Foa* race 2 resistance and susceptibility for GBS and for the data shown in Figures 2a and 3b,c were conducted in the greenhouse at 27–29°C as described previously (Epstein & Kaur, 2023; Kaur et al., 2022) (Methods S1). The GBS trial included 10 infested and 5 uninfested plants/accession, and the data shown in Figure 3a,d had 18 infested and 10 uninfested plants/cultivar; the plants were incubated in a completely randomized design. The null hypothesis that all cultivars in infested soil had an equivalent symptom severity was tested with nonparametric tests, either the Steel–Dwass multiple comparison test for all pairs or the Steel test for comparison of each accession to Challenger. Nonparametric tests were implemented in R via “coin.”

Determination of *Foa* race 2 and *Foa* race 4 conc in celery crowns was performed via real‐time quantitative PCR with race‐specific primers and PrimeTime nuclease probes with a 5′‐FAM reporter dye, an internal ZEN quencher and a 3′ Iowa black quencher (IDT, Coralville, IA) as described previously (Epstein & Kaur, 2023; Kaur et al., 2022). The data were analyzed via ANOVA and Tukey's HSD.

In the text, ± values are SEM.

## Author Contributions

LE and AVD originally conceived the study and obtained funding. SK and LE planned and conducted the greenhouse experiments. SK prepared the material for sequencing. DPH provided the genetic map. CL, JGM, LE, AVD, and PH planned the bioinformatic analyses. CL performed the bioinformatic analyses, visualizations, and data archiving. LE and CL cowrote the first draft. All the authors assisted with the writing and approved the final draft.

## Conflict Of Interest

The authors declare that they have no conflicts of interest.

## Consent for Publication

All authors consent to publication.

## Supporting information


**Figure S1.** Read mapping coverage of the Challenger genome.
**Figure S2.** Challenger genome survey.
**Figure S3.** An *A. graveolens* genetic map as a function of the physical position of each eSNP.
**Figure S4.** Count and length of structural variants between the Challenger and Ventura genomes.
**Figure S5.** The number of Challenger's 287 NLRs in each chromosome by NLR type.
**Figure S6.** Box plots of DESeq2‐normalized counts of 243 NLRs that were expressed in the Challenger crowns.

**Table S1.** The BUSCO (Benchmarking Universal Single‐Copy Orthologs) assessment of the Challenger genome and its annotation.
**Table S2.** The position and length of the centromeric regions in Challenger's chromosomes.
**Table S3.** Statistics about the assembly of each of cv. Challenger's 11 chromosomes.
**Table S4.** The genetic and physical maps of *Apium graveolens:* recombination rates (RR) across pseudochromosomes.
**Table S5.** Summary statistics of repetitive elements in the Challenger genome.
**Table S6.** The percentages of length, genes, LTRs, BUSCO genes, NLRs, and PRRs in the low‐recombination regions of each chromosome.
**Table S7.** *A. graveolens* accessions that were characterized by genotype‐by‐sequencing.
**Table S8.** Percent identity of genotype‐by‐sequencing SNP markers between pairs of 11 *A. graveolens* accessions.
**Table S9.** A comparison of the DNA similarity of Challenger's and Ventura's BUSCOs, NLRs, and PRRs.
**Table S10.** NLR‐Annotator's count of the number of sensor NLRs per chromosome in cvs. Challenger and Ventura.
**Table S11.** *P*‐values for differential expression of NLRs in Challenger crowns at 7 days post‐infestation.
**Table S12.** *P*‐values for differential expression of PRRs in Challenger crowns at 7 days post‐infestation.
**Table S13.** *A. graveolens* var. *rapaceum* with resistance to *Foa* race 2.
**Table S14.** Summary statistics about the PacBio HiFi reads from Challenger.
**Table S15.** Summary of samples for Challenger's PacBio Iso‐Seq sequencing.

**Data S1.** Predicted amino acid sequences of 40 464 genes in Challenger.
**Data S2.** NLRs in Challenger and their expression in crowns in mock and infested treatments.
**Data S3.** Potential pattern recognition receptors (PRRs) in Challenger and their expression in crowns in mock and infested treatments.

**Appendix S1.** Pathosystems in which resistance (R) genes to *F. oxysporum* have been molecularly identified.

**Methods S1.** Supporting Information.

## Data Availability

The data that support the findings of this study are openly available. The sequence and annotation data generated in this study have been deposited to the NCBI under the Bioproject PRJNA1203665. 3′QuantSeq data from Tall Utah were previously deposited to NCBI under Bioproject PRJNA591157 as accessions SRX7949416–SRX7949420 and SRX7949422 –SRX7949441. Other data are in this manuscript and the accompanying Supporting files. Any additional data and materials can be obtained by request.
