# Supplementary material for: A well‐annotated genome of Apium graveolens var. dulce cv. Challenger, a celery with resistance to Fusarium oxysporum f. sp. apii race 2
Source: Plant J. 2025 Jun 9;122(5):e70251. doi: 10.1111/tpj.70251 (PMC12148408; doi:10.1111/tpj.70251)
Supplement: Supplementary file 1 — Figure S1. Read mapping coverage of the Challenger genome. Figure S2. Challenger genome survey. Figure S3. An A. graveolens genetic map as a function of the physical position of each eSNP. Figure S4. Count and length of structural variants between the Challenger and Ventura genomes. Figure S5. The number of Challenger's 287 NLRs in each chromosome by NLR type. Figure S6. Box plots of DESeq2‐normalized counts of 243 NLRs that were expressed in the Challenger crowns. [file TPJ-122-0-s004.pdf]

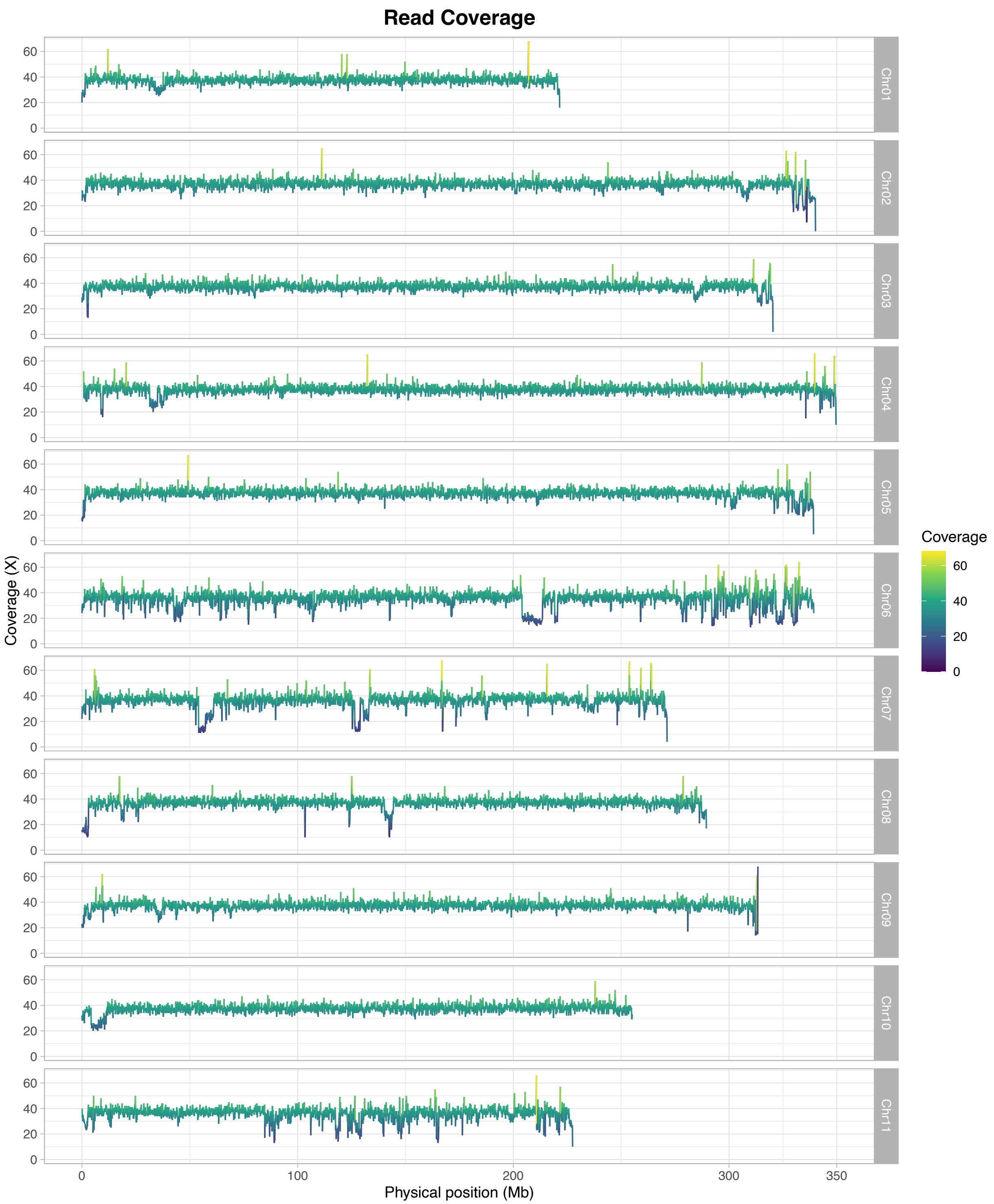

Fig. S1. Read mapping coverage of the Challenger genome. The read mapping coverage was calculated for each chromosome in 1 Mb windows.

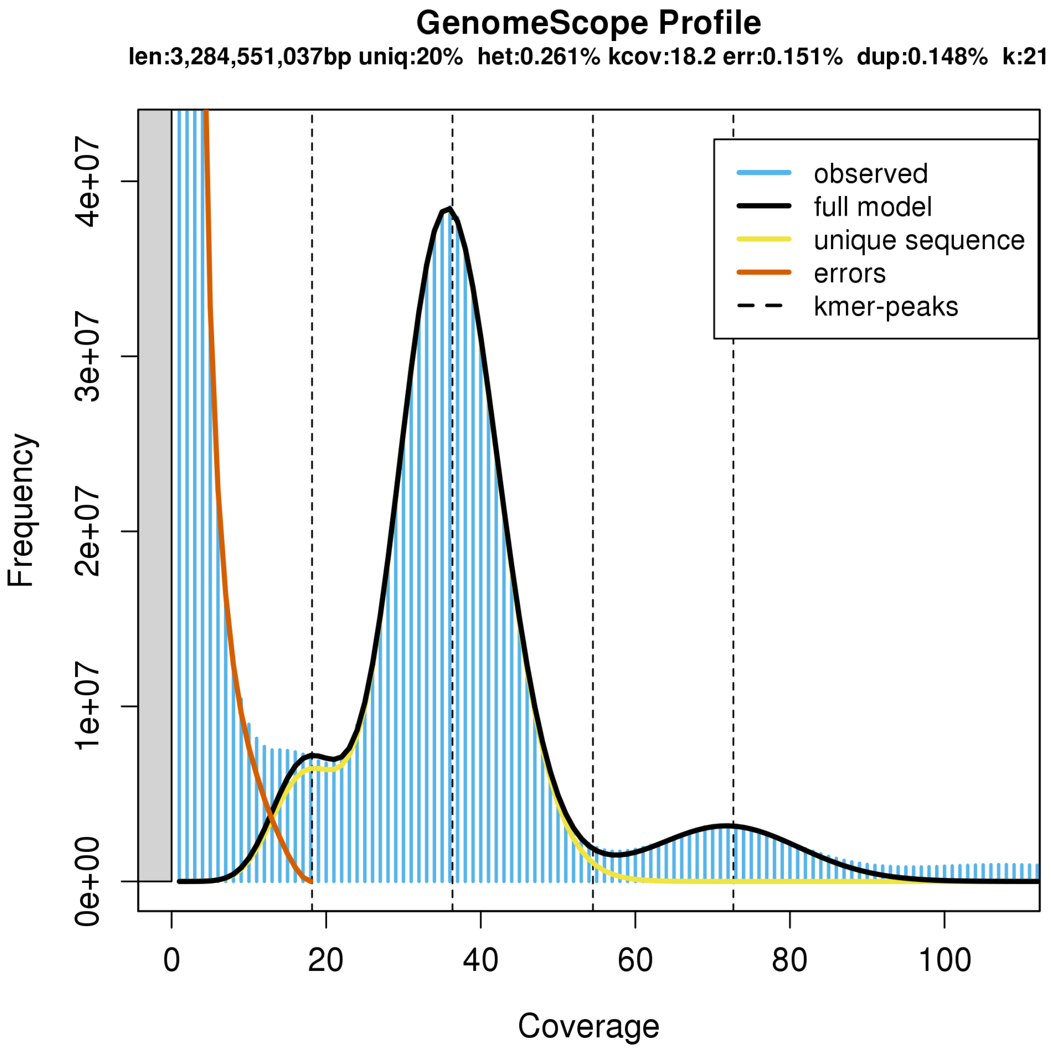

Fig. S2. Challenger genome survey. The estimation of genome size, heterozygosity, and repeat content were estimated with 21-mer frequency using jellyfish and visualized with GenomeScope. The coverage cutoff was increased to 1e6 due to high repetitiveness. len, haploid length; uniq, percentage of unique (non-repetitive) sequence; het, percentage of heterozygosity; err, percentage of sequencing error rate; dup, rate of mean read duplication.

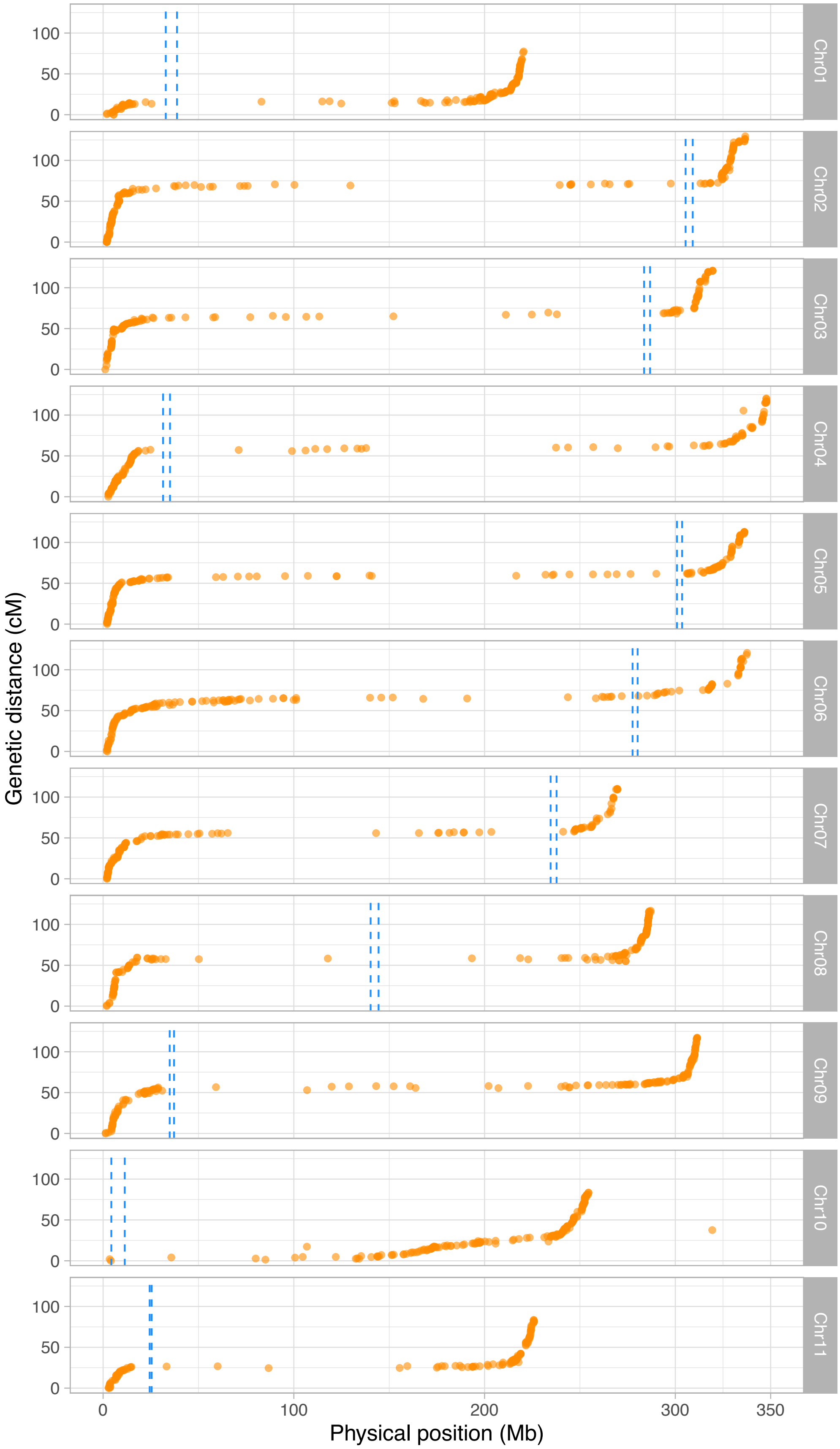

Fig. S3. An *A. graveolens* genetic map as a function of the physical position of each eSNP. The figure is a higher resolution image of the data shown in Fig. 1d. The blue dashed lines show the beginning and end of each centromeric region.

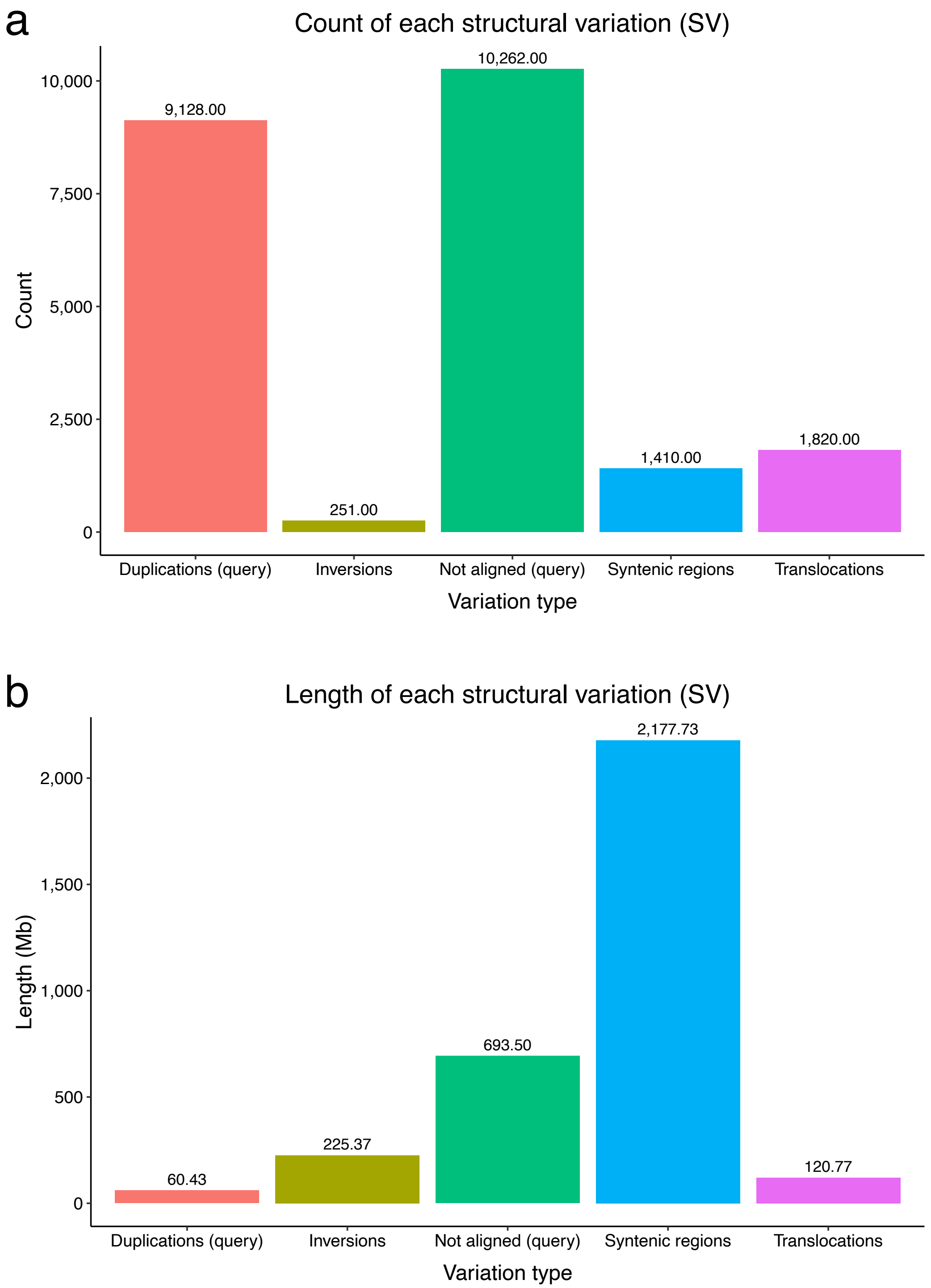

Fig. S4. Count and length of structural variants between the Challenger and Ventura genomes

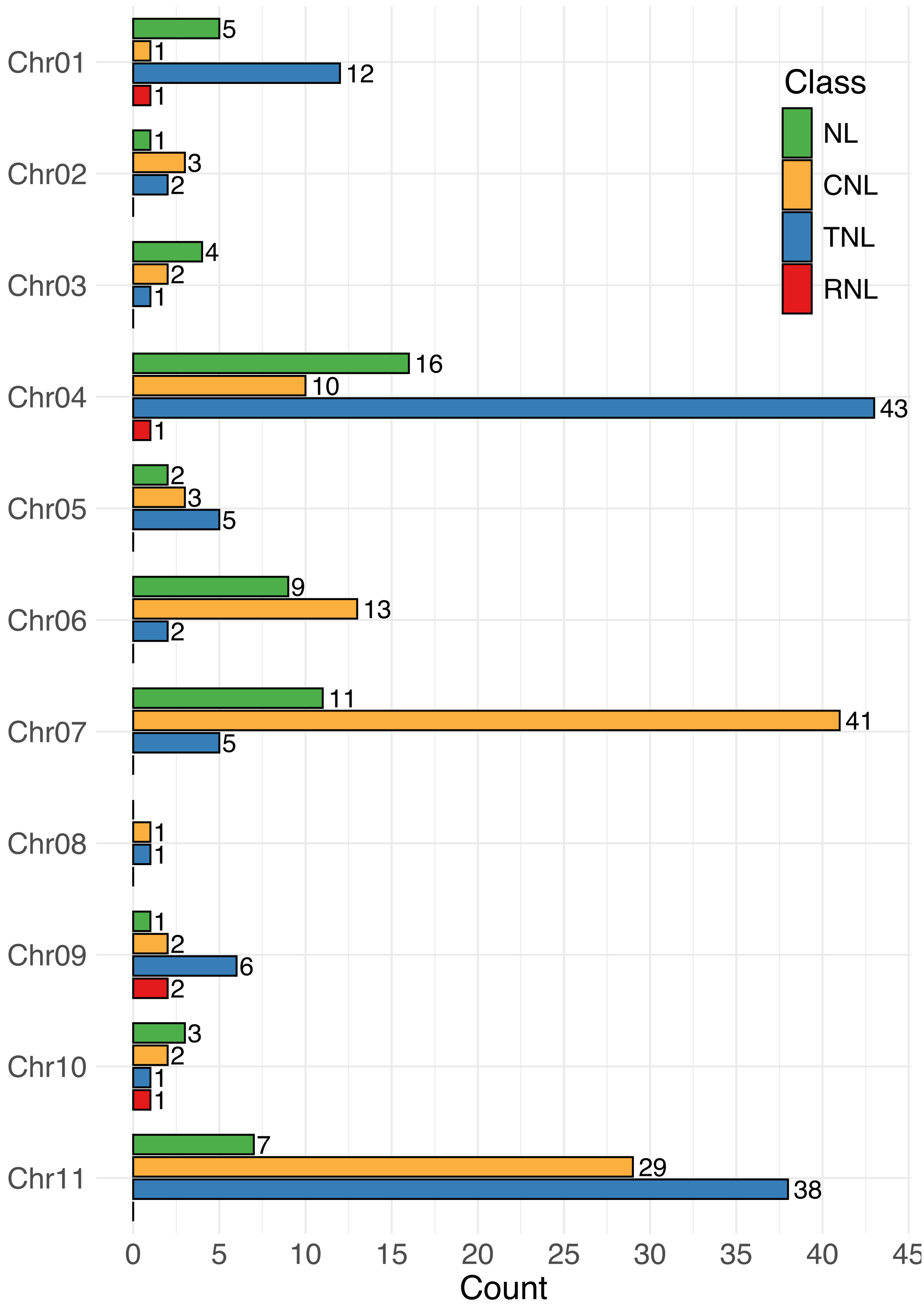

Fig. S5. The number of Challenger's 287 NLRs in each chromosome by NLR type.

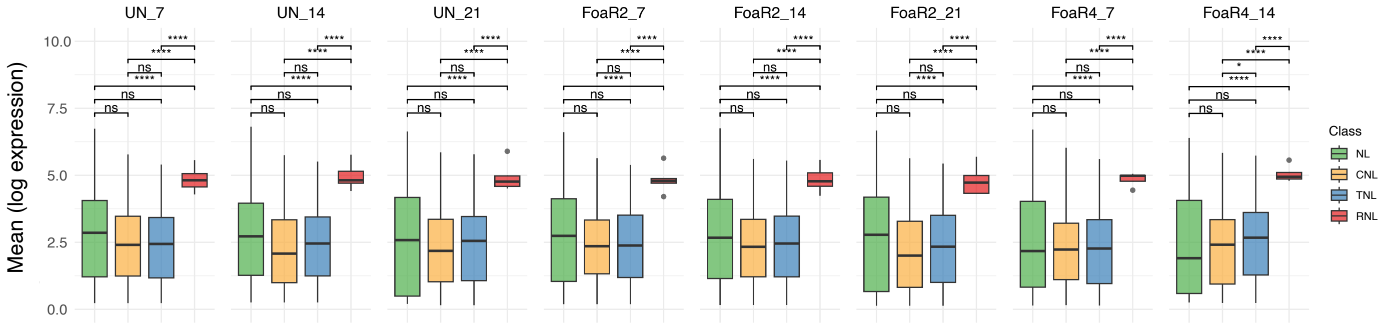

Fig. S6. Box plots of DESeq2-normalized counts of 243 NLRs that were expressed in the Challenger crowns. There were 42 NLs, 90 CNLs, 106 TNLs and 5 RNLs. Whiskers extend to 1.5 X the interquartile range. There were three pathogen treatments: UN, mock-infested; and infested with either *Foa* race 2 (FoaR2) or Foa race 4 (FoaR4). All the plants in the treatments were sampled destructively at 7- and 14- days post infestation/transplantation, but only the uninfested plants and those in the soil infested with *Foa* race 2 were sampled at 21 dpi because Challenger in *Foa* race 4 had started to die. Each replicate was a pool of 5 plants. There were 3 and 4 replicates for the uninfested and infested treatments, respectively, except for 3 replicates for the *Foa* race 2 treatment at 7 dpi. Significance at $\leq$0.0001 (***), $\leq$0.01 (**) or $\leq$0.05 (*) was determined by the Wilcoxon nonparametric test, adjusted for multiple comparisons.
