# Supplementary material for: A well‐annotated genome of Apium graveolens var. dulce cv. Challenger, a celery with resistance to Fusarium oxysporum f. sp. apii race 2
Source: Plant J. 2025 Jun 9;122(5):e70251. doi: 10.1111/tpj.70251 (PMC12148408; doi:10.1111/tpj.70251)
Supplement: Supplementary file 4 — Appendix S1. Pathosystems in which resistance (R) genes to F. oxysporum have been molecularly identified. [file TPJ-122-0-s002.docx]

Lee et al. 2025. The Plant Journal

**Supporting Information Appendix S1:** Pathosystems in which resistance (R) genes to *F. oxysporum* have been molecularly identified**.**

In crops resistant to *F. oxysporum*, NLRs have been identified in five cases. In melon, the R gene *Fom-2* gene encodes an NLR with an atypical CC-NB-LRR that confers race-specific resistance (Joobeur *et al.*, 2004). *Fom-1* encodes a TIR-NB-LRR protein (Brotman *et al.*, 2013). The cabbage gene *FOC1* apparently encodes a TIR-NB-LRR and presumably confers resistance to *F. oxysporum* f. sp. *conglutinans* race 1 (Lv *et al.*, 2014). In tomato, the R gene *I-2* encodes an NLR with a CC-NB-LRR (Simons *et al.*, 1998). The banana gene RGA2, which is a CC-NB-LRR, may be an important gene for resistance to *F. oxysporum* f. sp. *cubense* race 1 (Dale *et al.*, 2017).

Two *I* genes that are introgressed from *Solanum pennelii* can induce resistance in tomato depending on the pathogen race: the *I-3* gene encodes an RLK with a transmembrane protein, an extracellular S-receptor domain, an intracellular serine/threonine kinase domain and a G-type lectin domain (Catanzariti *et al.*, 2015), and the *I-7* gene encodes an RLP with extracellular leucine-rich repeats and short cytoplasmic receptor-like domains (Gonzalez-Cendales *et al.*, 2016). Another *I* gene, also called *I*-1, was introgressed from *S. pimpinellifolium* and encodes an RLP with extracellular leucine-rich repeats (Catanzariti *et al.*, 2017). In Arabidopsis, *RFO3* (**r**esistance to ***F****.* ***o****xysporum* f. sp. *matthioli)* encodes for an RLK with a lectin receptor-like kinase (Cole and Diener, 2013)); the RLKs *RLK7* and *MIK2* confer resistance to *F. oxysporum* and other pathogens (Hou *et al.*, 2019; Hou *et al.*, 2021). The Arabidopsis RLK gene *WAKL22* (*RFO1*) encodes a wall-associated receptor-like kinase domain and a second kinase domain and confers resistance to multiple *f.* spp. that infect Arabidopsis (Diener and Ausubel, 2005; Huerta *et al.*, 2023).

**Brotman, Y., Normantovich, M., Goldenberg, Z., et al.** (2013) Dual resistance of melon to *Fusarium oxysporum* races 0 and 2 and to Papaya ring-spot virus is controlled by a pair of head-to-head-oriented NB-LRR genes of unusual architecture. *Molecular Plant*, **6**, 235–238.

**Catanzariti, A., Do, H.T.T., Bru, P., Sain, M., Thatcher, L.F., Rep, M. and Jones, D.A.** (2017) The tomato *I* gene for Fusarium wilt resistance encodes an atypical leucine‐rich repeat receptor‐like protein whose function is nevertheless dependent on SOBIR 1 and SERK 3/ BAK 1. *Plant J*, **89**, 1195–1209.

**Catanzariti, A.M., Lim, G.T.T. and Jones, D.A.** (2015) The tomato *I-3* gene: a novel gene for resistance to Fusarium wilt disease. *New Phytologist*, **207**, 106–118.

**Cole, S.J. and Diener, A.C.** (2013) Diversity in receptor‐like kinase genes is a major determinant of quantitative resistance to *Fusarium oxysporum* f.sp. *matthioli*. *New Phytol*, **200**, 172–184.

**Dale, J., James, A., Paul, J.-Y., et al.** (2017) Transgenic Cavendish bananas with resistance to Fusarium wilt tropical race 4. *Nat Commun*, **8**, 1496.

**Diener, A.C. and Ausubel, F.M.** (2005) *RESISTANCE TO FUSARIUM OXYSPORUM 1* , a dominant Arabidopsis disease-resistance gene, Is not race specific. *Genetics*, **171**, 305–321.

**Epstein, L. and Kaur, S.** (2023) *Apium graveolens* PI 181714 is a source of resistance to *Fusarium oxysporum* f. sp. *apii* race 4 in celery (*A. graveolens* var. *dulce*). *Plant Breeding*, **142**, 109–117.

**Gíslason, M.H., Nielsen, H., Almagro Armenteros, J.J. and Johansen, A.R.** (2021) Prediction of GPI-anchored proteins with pointer neural networks. *Current Research in Biotechnology*, **3**, 6–13.

**Gonzalez-Cendales, Y., Catanzariti, A.-M., Baker, B., Mcgrath, D.J. and Jones, D.A.** (2016) Identification of *I* *-7* expands the repertoire of genes for resistance to Fusarium wilt in tomato to three resistance gene classes: Tomato *I-7* gene for Fusarium wilt resistance. *Molecular Plant Pathology*, **17**, 448–463.

**Henry, P., Kaur, S., Pham, Q.A.T., Barakat, R., Brinker, S., Haensel, H., Daugovish, O. and Epstein, L.** (2020) Genomic differences between the new *Fusarium oxysporum* f. sp. *apii* (Foa) race 4 on celery, the less virulent Foa races 2 and 3, and the avirulent on celery f. sp. *coriandrii*. *BMC Genomics*, **21**, 730.

**Hou, S., Liu, D., Huang, S., et al.** (2021) The Arabidopsis MIK2 receptor elicits immunity by sensing a conserved signature from phytocytokines and microbes. *Nat Commun*, **12**, 5494.

**Hou, S., Shen, H. and Shao, H.** (2019) PAMP-induced peptide 1 cooperates with salicylic acid to regulate stomatal immunity in *Arabidopsis thaliana*. *Plant Signaling & Behavior*, **14**, 1666657.

**Huerta, A.I., Sancho-Andrés, G., Montesinos, J.C., et al.** (2023) The WAK-like protein RFO1 acts as a sensor of the pectin methylation status in Arabidopsis cell walls to modulate root growth and defense. *Molecular Plant*, **16**, 865–881.

**Joobeur, T., King, J.J., Nolin, S.J., Thomas, C.E. and Dean, R.A.** (2004) The fusarium wilt resistance locus *Fom‐2* of melon contains a single resistance gene with complex features. *The Plant Journal*, **39**, 283–297.

**Kaur, S., Barakat, R., Kaur, J. and Epstein, L.** (2022) The effect of temperature on disease severity and growth of *Fusarium oxysporum* f. sp. *apii* races 2 and 4 in celery. *Phytopathology*, **112**, 364–372.

**Lohman, B.K., Weber, J.N. and Bolnick, D.I.** (2016) Evaluation of TagSeq, a reliable low-cost alternative for RNAseq. *Molecular Ecology Resources*, **16**, 1315–1321.

**Lv, H., Fang, Z., Yang, L., et al.** (2014) Mapping and analysis of a novel candidate Fusarium wilt resistance gene *FOC1* in *Brassica oleracea*. *BMC Genomics*, **15**, 1094.

**Ødum, M.T., Teufel, F., Thumuluri, V., Almagro Armenteros, J.J., Johansen, A.R., Winther, O. and Nielsen, H.** (2024) DeepLoc 2.1: multi-label membrane protein type prediction using protein language models. *Nucleic Acids Research*, **52**, W215–W220.

**Simons, G., Groenendijk, J., Wijbrandi, J., Reijans, M., Groenen, J. and Diergaarde, P.** (1998) Dissection of the Fusarium *I2* gene cluster in tomato reveals six homologs and one active gene copy. *The Plant Cell*, **10**, 1055–1068.
