# Supplementary material for: A well‐annotated genome of Apium graveolens var. dulce cv. Challenger, a celery with resistance to Fusarium oxysporum f. sp. apii race 2
Source: Plant J. 2025 Jun 9;122(5):e70251. doi: 10.1111/tpj.70251 (PMC12148408; doi:10.1111/tpj.70251)
Supplement: Supplementary file 5 — Methods S1. Supporting Information. [file TPJ-122-0-s001.docx]

Lee et al. 2025. The Plant Journal

**Supporting experimental procedures: Methods S1**

Plants and fungi. Celery was obtained from the following sources: celery cvs. Challenger and Sabroso (Syngenta, Downers Grove, IL); Command and Ventura (White Seed Co., Oxnard, CA); Tall Utah 52-70R Improved (W. Atlee Burpee & Co, Warminster Township, PA); and Tendercrisp (Ferry-Morse, Norton, MA). The celery breeding line UC390S was obtained from the University of California (UC) Davis *A. graveolens* germplasm collection. The following *A. graveolens* were obtained from the USDA collection (<https://www.ars.usda.gov/northeast-area/geneva-ny/plant-genetic-resources-unit-pgru/docs/celery-collection/>) and multiplied at UC Davis: celeriac accessions PI 169001, PI 176419, PI 261810 and PI 320912; and *A. graveolens* PI 181714.

Samples for IsoSeq. Challenger seeds were surface sterilized and then germinated axenically on moist filter paper in the laboratory for 15 days. Three of the samples were collected from young axenic tissues: germinable seeds, i.e., five days after surface sterilization and just before germ tube emergence; 15-day-old seedling roots from below the point of seed attachment; and 15-day-old seedling stems and first leaves from above the point of seed attachment. Nine samples were collected from tissues of Challenger plants that were grown in the greenhouse (Kaur *et al.*, 2022): leaves of various ages, from just unfurled to fully expanded but not senescent; petioles of various ages from very young to 1 cm diam; crowns 2.25, 2.5 and 2.75 months after seed germination and approximately 1 cm long; vegetative buds; unopened flower buds; open flowers before fertilization; flowers ca. one week after fertilization; young, green seeds ca. three to five weeks after fertilization; and mature seeds from storage. After harvest, each of these tissues was quickly placed in prechilled aluminum foil, frozen in liquid N_2,_ and then extracted using RNAeasy (Qiagen, Redwood City, USA) and indexed for one Iso-Seq library.

Three additional tissues were collected for a second Iso-Seq library: two-month-old seedlings in potting soil were gently washed to remove the potting medium, and the subterranean tissue was incubated in a suspension of 7.6 × 10^6^ *F. oxysporum* f. sp. *apii* race 2 conidia in 0.1% water agar for 10 min and then transplanted into tubes (Kaur *et al.*, 2022) and incubated in a greenhouse for 7, 14 or 21 days post-transplantation/infestation. After harvest, the crowns were quickly washed free of soil and blotted dry. After the roots were removed, each of the 10 crowns per sampling time, approximately 1 cm in length, was placed in prechilled aluminum foil, frozen in liquid N_2_ and then extracted and indexed for the second Iso-Seq library. As a check, the roots and crowns of 10 additional samples/time points that were inoculated or not were examined for symptoms of vascular discoloration.

Samples for Tag-Seq. Additional crown tissue was also collected for Tag-Seq (Lohman *et al.*, 2016). In a greenhouse trial with a cv. Challenger with a completely randomized design, two-month-old Challenger seedlings in potting soil were gently washed free of potting medium, and the subterranean tissue was incubated for 10 min in either a mock-inoculated control of 0.1% water agar or a suspension of 7.6 × 10^6^ conidia/ml 0.1% water agar and then transplanted into tubes with a perlite–greenhouse soil mixture (Kaur *et al.*, 2022). There were 3 pathogen treatments (uninfested mock, infested with *Foa* race 2, and infested with *Foa* race 4) and either 2 or 3 sampling times; all the treatments were sampled destructively at 7- and 14- days post infestation, but only the uninfested plants and those in the soil infested with *Foa* race 2 were sampled at 21 dpi because Challenger in *Foa* race 4 had started to die. Each replicate was a pool of 5 plants. There were 3 and 4 replicates for the uninfested and infested treatments, respectively, except for 3 replicates for the *Foa* race 2 treatment at 7 dpi.

After the soil was removed, each of the 5 plants/replicate were processed rapidly; the 1 to 3 roots from the crown were removed, and each crown (ca. 1 cm in length) was cut longitudinally in half; one half was processed for Tag-Seq, and one half was processed for DNA extraction for quantification of fungal biomass as described below. For Tag-Seq, crowns were crushed with a sterilized, precooled pestle while keeping everything on ice, transferred into precooled aluminum foil, wrapped and immediately flash frozen in liquid N_2_ and then stored at -80 °C.

There was an independent greenhouse trial with cv. Tall Utah 52-70R Improved. Methods were reported by Henry et al. (Henry *et al.*, 2020), based on Lohman et al. (Lohman *et al.*, 2016). For each of the five replicates, eight celery cultivar Tall Utah 52–70 R Improved plants were either transplanted into uninfested soil or soil infested with either *Foa* race 2 or *Foa* race 4 and incubated for 21 days.

Assays for symptoms and either *Foa* race 2 or race 4 biomass in tissue. The bioassays for *Foa* race 2 resistance and susceptibility to GBS and for the data shown in Figs. 2a and 3b-c were conducted in the greenhouse at 27-29 °C as described previously (Epstein and Kaur, 2023; Kaur *et al.*, 2022). Briefly, each two-month-old seedling was transplanted as a plug into a tube that contained a perlite and potting mixture that was either uninfested or infested with millet that was colonized by the indicated *Foa* strain. After 2 months, the washed roots were scored on the basis of the following ordinal symptomatic score: 0, asymptomatic; 1, discoloration limited to the fine roots; 2, discoloration in the main roots but not in the crown; 3, discoloration in the crown but on < ¼ of the circumference of the vasculature; 4, discoloration in > ¼ of the circumference of the vasculature; and 5, plant death.

Selection of potential pattern recognition receptors. We first used DRAGO 3 in PRGDb 4.0 to select a total of 566 putative PRRs in the following categories: using their abbreviations, receptor-like kinase with leucine-rich repeats (RLK), receptor-like protein with leucine-rich repeats (RLP), receptor-like kinase with a LysM domain (LYK), receptor-like protein with a LysM domain (LYS) and kinase with a lectin domain (LECRK). We then checked these proteins with DeepLoc 2.1 (Ødum *et al.*, 2024) to determine if all of these genes were independently categorized as localized in the cell membrane; DeepLoc classified only 350 as in the cell membrane, with the remainder either in intracellular membranes, cytoplasmic or extracellular. We then used keyword searches of our annotation files, followed by DeepLoc 2.1 and NetGPI (Gíslason *et al.*, 2021), and identified an additional 140 that were not among our original PRRs, for a count of 490 PRRs that are most likely localized in the cell membrane (Table 3 in the main text, Data S3).

**Epstein, L. and Kaur, S.** (2023) *Apium graveolens* PI 181714 is a source of resistance to *Fusarium oxysporum* f. sp. *apii* race 4 in celery (*A. graveolens* var. *dulce*). *Plant Breeding*, **142**, 109–117.

**Gíslason, M.H., Nielsen, H., Almagro Armenteros, J.J. and Johansen, A.R.** (2021) Prediction of GPI-anchored proteins with pointer neural networks. *Current Research in Biotechnology*, **3**, 6–13.

**Henry, P., Kaur, S., Pham, Q.A.T., Barakat, R., Brinker, S., Haensel, H., Daugovish, O. and Epstein, L.** (2020) Genomic differences between the new *Fusarium oxysporum* f. sp. *apii* (Foa) race 4 on celery, the less virulent Foa races 2 and 3, and the avirulent on celery f. sp. *coriandrii*. *BMC Genomics*, **21**, 730.

**Kaur, S., Barakat, R., Kaur, J. and Epstein, L.** (2022) The effect of temperature on disease severity and growth of *Fusarium oxysporum* f. sp. *apii* races 2 and 4 in celery. *Phytopathology*, **112**, 364–372.

**Lohman, B.K., Weber, J.N. and Bolnick, D.I.** (2016) Evaluation of TagSeq, a reliable low-cost alternative for RNAseq. *Molecular Ecology Resources*, **16**, 1315–1321.

**Ødum, M.T., Teufel, F., Thumuluri, V., Almagro Armenteros, J.J., Johansen, A.R., Winther, O. and Nielsen, H.** (2024) DeepLoc 2.1: multi-label membrane protein type prediction using protein language models. *Nucleic Acids Research*, **52**, W215–W220.
